# Microglia–Neuron Crosstalk: An Intimate Molecular Conversation in Neurodegeneration

**DOI:** 10.3390/ijms27042011

**Published:** 2026-02-20

**Authors:** Shiqi Wang, Sichen Wang, Hongzhuan Chen, Jianrong Xu

**Affiliations:** 1 The Research Center for Traditional Chinese Medicine, Shanghai Institute of Infectious Diseases and Biosecurity, School of Integrative Medicine, Shanghai University of Traditional Chinese Medicine, Shanghai 201203, China; wsq496303154@126.com (S.W.); wsc1736472769@163.com (S.W.); 2 Future Health Laboratory, Shuguang Hospital Affiliated to Shanghai University of Traditional Chinese Medicine, Shanghai 201203, China; 3 Institute of Interdisciplinary Integrative Medicine Research, Shanghai University of Traditional Chinese Medicine, Shanghai 201203, China

**Keywords:** microglia, neuron, crosstalk, inflammation, neurodegenerative disease, bioenergetics

## Abstract

Microglia are a unique cell population in the central nervous system (CNS) and serve as its resident immune cells. They have long been recognized for their critical contributions to CNS development and the maintenance of neuronal network health, particularly in the context of neuroprotection against neurodegenerative diseases. However, the mechanisms by which microglia interact with and influence neurons have remained largely unclear. Recent advances in genetics, pharmacology, and imaging technologies have begun to unveil the mechanisms underlying microglia–neuron communication. Here, from the perspective of microglia, we review the diverse direct and indirect pathways and key molecules through which microglia interact with neurons under both physiological and pathological conditions. This rapidly expanding knowledge is reshaping our understanding of neuron–glia physiology and pathology in neurodegenerative diseases.

## 1. Introduction

Microglia (MG), the primary immune sentinels of the central nervous system (CNS), continuously monitor neuronal health to maintain neural networks and support normal brain function. Microglia are widely distributed throughout the brain, comprising 5–12% of total glial cells across different regions [1,2]. They exhibit remarkable spatial and temporal heterogeneity, with their phenotype dictated by the local microenvironment and finely tuned to niche-specific demands. In the adult brain, microglia possess highly ramified processes with small cell bodies and maintain their population through self-renewal.

Microglia engage in complex bidirectional communication with neurons, which is essential for brain development, homeostasis maintenance, and resolution of CNS inflammation. During brain development, microglia guide circuit formation [3], participate in synapse formation and pruning [4], support neurogenesis and maintain neural networks [5]. Additionally, microglia contribute to injury repair, regulate vascular function and cerebral blood flow [6], and even directly modulate sympathetic nervous output [7]. As the principal signaling units of the nervous system, neurons actively modulate microglial function and refine circuit connectivity through the continuous release of diverse signals—ranging from membrane-bound proteins and soluble factors to metabolic byproducts [8]. This form of bidirectional regulation underpins all phases of brain development, homeostatic maintenance, and pathological adaptation, establishing a finely tuned signaling axis.

In recent years, neuroimmunometabolism has emerged as an interdisciplinary field focusing on the convergence of immune and metabolic cascades within the CNS. This metabolic plasticity involves the exchange of energy substrates (e.g., ATP/ADP, glucose, fatty acids, and glutamine) and processes such as mitochondrial transfer, constituting a dominant mechanism of crosstalk [9]. This field has uncovered unique bioenergetic interactions between microglia and neurons.

Depletion, dysfunction, or aberrant activation of microglia represent a key driver of neurodegenerative diseases. Aberrantly activated microglia release excessive pro-inflammatory cytokines and neurotoxic molecules, including reactive oxygen species (ROS) and nitric oxide (NO), creating a hostile microenvironment that damages neurons and exacerbates neurodegeneration [10,11]. Damaged neurons may release damage-associated molecular patterns (DAMPs), which activates microglia and establishes a vicious cycle. This dysregulated intercellular communication results in neuronal loss and dysfunction, ultimately manifesting as cognitive and motor deficits [12]. However, the mechanisms by which microglia regulate this crosstalk in neurodegenerative diseases—such as Alzheimer’s disease (AD), Parkinson’s disease (PD), and multiple sclerosis (MS)—particularly around pathological hallmarks like amyloid plaques, remain poorly understood.

Current research has comprehensively reviewed direct and indirect modes of microglia–neuron crosstalk, which bidirectionally regulate neural development, synaptic plasticity, and metabolic homeostasis. However, a critical gap remains in understanding the precise molecular mechanisms through which microglia modulate these interactions specifically within the context of neurodegenerative diseases, particularly in relation to key pathological features like amyloid plaques in AD.

**Figure 1:**
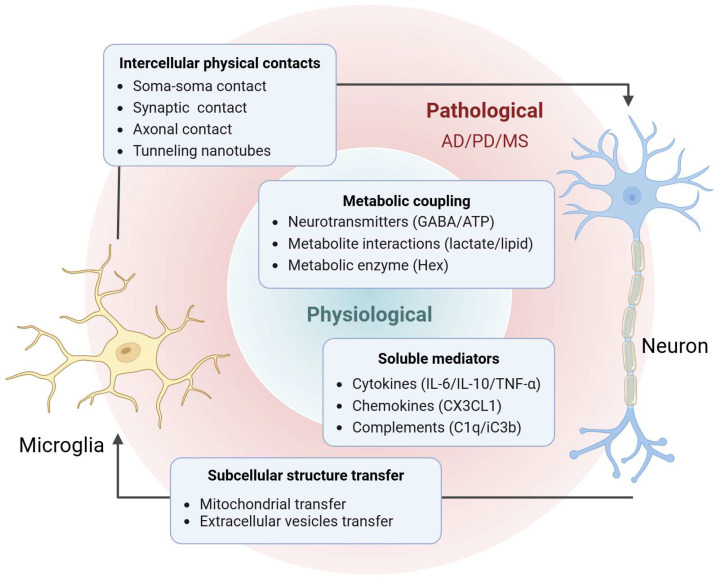
The Crosstalk Mechanism between Microglia and Neurons.

To clarify this complex intercellular dialogue, this review delineates the unidirectional regulatory roles of microglia on neurons and of neurons on microglia. Building on this foundation, further focus is placed on the reciprocal communication networks jointly established by microglia and neurons. In addition, we summarize the evolving mechanisms and therapeutic implications of such crosstalk in neurodegenerative diseases, thereby providing a systematic conceptual foundation for future interventions targeting the neuroimmune–metabolic axis (Figure 1).

## 2. The Physiological and Pathological Roles of Microglia

Microglia are tissue-resident macrophages (TRMs) of the brain parenchyma, originating from the yolk sac during embryogenesis and entering and colonizing the CNS parenchyma early in embryonic development [13]. Microglia dynamically survey their surroundings by constantly extending and retracting motile processes, thereby performing immune surveillance [14]. Additionally, they maintain their survival through basal autophagy and clear apoptotic cell debris via phagocytosis [15]. As the resident mononuclear phagocytes of the CNS, adult microglia are sustained primarily through local self-renewal, with limited contribution from peripheral monocytes under physiological conditions. By performing diverse functions, microglia contribute to homeostasis and host defense, displaying distinct transcriptional programs and morphological states depending on environmental and physiological contexts [16]. Microglia are equipped with a unique set of sensing molecules collectively termed the “sensome”. This system enables them to detect subtle microenvironmental changes, rapidly migrate to sites of injury, and execute repair and defense functions [17].

The functional diversity of microglia is fundamentally rooted in their heterogeneity across spatial, temporal, and contextual dimensions. The traditional M1/M2 classification, which categorizes activated microglia into pro-inflammatory/neurotoxic (M1) and anti-inflammatory/neuroprotective (M2) phenotypes, is now widely regarded as an oversimplified and misleading framework. Such binary and inflexible classifications promote a reductive “good versus bad” perspective on microglial function and can hinder scientific progress in the field [18]. Evidence indicates that microglia in vivo do not segregate into these distinct polarized categories [19]. Advances in single-cell and multi-omics technologies have unveiled a diverse continuum of microglial states that extend far beyond the M1/M2 paradigm. For instance, single-cell RNA sequencing has identified context-dependent states such as disease-associated microglia (DAM), which is a triggering receptor expressed on myeloid cells 2 (TREM2)-dependent transcriptional state commonly observed in neurodegenerative conditions [20,21], along with other distinct profiles including the microglial neurodegenerative phenotype (MGnD) [22,23] and lipid-droplet-accumulating microglia (LDAM) [24]. These states are not fixed subtypes but are dynamic, plastic, and often transitional, reflecting the capacity of microglia to adapt to local environmental signals. Consequently, the functional diversity of microglia is shifting toward a multidimensional descriptive framework that integrates molecular signatures with direct functional validation, moving beyond outdated dichotomous nomenclature.

Under physiological conditions, microglia perform key functions including immune surveillance, synaptic pruning, and maintenance of microenvironmental homeostasis. They recognize pathogen-associated molecular patterns (PAMPs) and DAMPs to maintain homeostasis and mount host defense responses. Binding of PAMPs or DAMPs to microglial surface receptors—such as toll-like receptors (TLRs), NOD-like receptors (NLRs), and C-type lectin receptors (CLRs)—triggers downstream signaling cascades, resulting in microglial activation and initiation of immune responses [25]. The brain, one of the most energy-demanding organs, accounts for approximately 20% of total body energy consumption. Glucose serves as its primary energy source, metabolized via glycolysis in the cytoplasm and oxidative phosphorylation (OXPHOS) in mitochondria to generate ATP [26]. Microglia express transporters for various energy substrates, including glucose, fatty acids, and glutamine. Under homeostatic conditions, they continuously monitor the brain microenvironment and adapt their metabolic pathways in response to metabolic cues. In homeostasis, microglia predominantly rely on OXPHOS to meet energy demands, supporting baseline functions such as debris clearance and apoptotic cell removal. However, under glucose scarcity, they rapidly switch to alternative substrates like glutamine or lactate to preserve adaptability and surveillance capacity [27].

Under pathological conditions, microglial functions undergo profound changes. During neuroinflammation, they reprogram their metabolism from OXPHOS to aerobic glycolysis—a phenomenon reminiscent of the Warburg effect observed in cancer cells. Although glycolysis yields far less ATP (2 per glucose molecule versus ~36 via OXPHOS), it enables rapid energy production to support inflammatory functions, including pathogen and debris clearance [28]. Concomitantly, the pentose phosphate pathway (PPP) and fatty acid synthesis are upregulated, fueling the production of pro-inflammatory cytokines and ROS [29,30]. Persistent inflammatory activation disrupts metabolic homeostasis, establishing a self-sustaining cycle of tissue damage. ROS and other neurotoxic molecules released by microglia create a hostile microenvironment that exacerbates neuronal injury. In neurodegenerative diseases, this functional shift frequently transitions from protective to detrimental. For instance, in DAM, dysregulated TREM2-dependent lipid metabolism may exacerbate protein aggregate accumulation [31]. Moreover, microglia engage in bidirectional crosstalk with neurons via signaling pathways involving cytokines, neurotransmitters, neuropeptides, and select metabolites. This complex communication requires substantial energy expenditure and relies on efficient mitochondrial metabolism [32].

**Figure 2:**
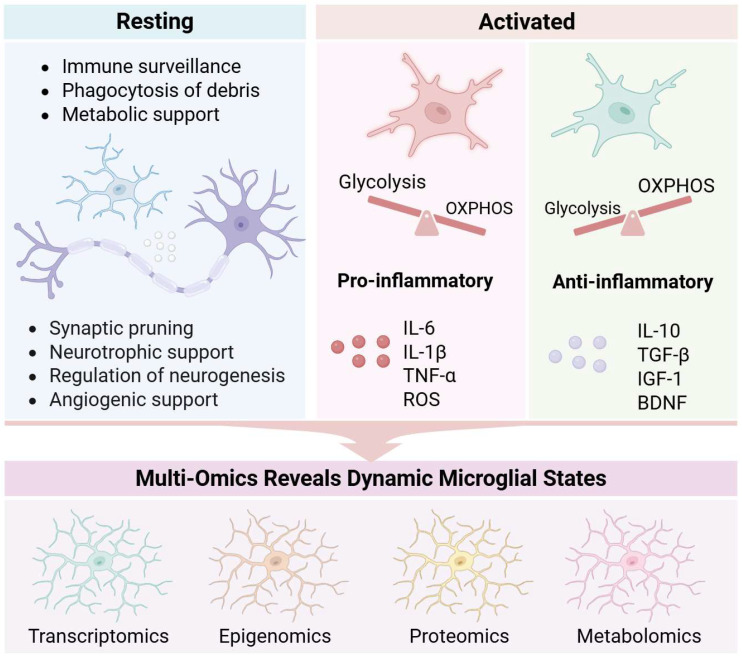
Microglia in Physiological and Pathological States.

In summary, microglial functions—from ontogeny and activation states to metabolic adaptability—constitute a highly dynamic system. Under physiological conditions, they preserve CNS homeostasis; under pathological conditions, metabolic reprogramming can amplify inflammatory responses. These insights lay the foundation for exploring microglia–neuron metabolic crosstalk (Figure 2).

## 3. Microglial Regulation of Neurons: From Chemical Signals to Physical Contacts

### 3.1. Cytokine-Mediated Microglia-to-Neuron Signaling

Microglia sense microenvironmental changes and modulate neuronal function primarily through cytokine release. For instance, activation of microglial P2X_4_ receptors by ATP induces a rapid rise in intracellular Ca^2+^, triggering the release of cytokines such as IL-1β and TNF-α, which enhance synaptic transmission within spinal pain pathways [33]. Subsequent studies have further shown that P2X_4_-activated microglia release brain-derived neurotrophic factor (BDNF). By acting on neuronal TrkB receptors, BDNF reverses the anion gradient and disinhibits pain-transmitting neurons, which is a key mechanism underlying pain hypersensitivity [34]. This work represents a pivotal shift in neuropathic pain research, redirecting the focus from purely “neuronal dysfunction” to “neuroimmune communication.” Beyond modulating synaptic transmission, activated microglia influences neuronal integrity through multiple pathways. Pro-inflammatory TNF-α secreted by activated microglia inhibits the AMP-activated protein kinase (AMPK)/sirtuin 3 (SIRT3) pathway, thereby inducing neuronal mitochondrial dysfunction [1,2]. Microglia also indirectly regulate neuronal survival by inducing reactive astrocyte phenotypes. Specifically, microglia-derived IL-1α, TNF-α trigger the formation of neurotoxic A1 astrocytes characterized by robust C3 upregulation [35]. Unlike neuroprotective A2 astrocytes, A1 astrocytes exhibit impaired synapse-forming capacity and secrete potent neurotoxic factors [36]. The NLRP3 inflammasome plays a central role in driving pro-inflammatory cytokine release from activated microglia. Sustained NLRP3 activation amplifies both phagocytic activity and inflammatory cytokine production, thereby exacerbating neuropathology (e.g., lumbar disc degeneration) [37].

Conversely, microglia can mount protective responses. In alternative activation states, they secrete anti-inflammatory cytokines (IL-4, IL-10) and neurotrophic factors that promote neuronal repair, neuroprotection, and CNS homeostasis [38]. Additionally, microglia induce neuronal IL-6 production; in chronic non-resolving inflammation, this neuron-derived IL-6 forms complexes with soluble IL-6R to ameliorate learning deficits and stimulate neurogenesis [39]. In human neural-immune organoid models (hMGEOs), microglia-derived insulin-like growth factor 1 (IGF1) also can enhance progenitor proliferation and GABAergic neuron differentiation [40]. In spinal cord injury, IL-4 delivery promotes M2 polarization and functional recovery, while inhibition of IL-6 or IL-17 reduces M1-associated neurotoxicity [41,42,43]. Thus, cytokines act both as effector molecules and as key regulators of the microglial phenotype.

### 3.2. Receptor- and Complement-Mediated Microglia–Neuron Communication

Microglia express diverse receptors that critically regulate neuronal function (Table 1). During critical developmental periods in mice, gamma-aminobutyric acid (GABA) receptor-expressing microglia selectively prune inhibitory synapses (but not excitatory ones) by activating a transcriptional synaptic remodeling program, thereby modulating neuronal activity and animal behavior [44]. Furthermore, the overexpression of TREM2 enhances dendritic spine engulfment by microglia, thereby ameliorating morphological abnormalities and cognitive deficits [45]. Complement receptor 3 (CR3) recognizes iC3b and mediates microglial adhesion, chemotaxis, and phagocytosis. Blocking CR3 attenuates dopaminergic neuron loss in MPTP- and LPS-induced mouse models [46,47]. High CR3 expressions also drive p47phox phosphorylation and membrane translocation, thereby activating NADPH oxidase 2 (NOX2). The resulting superoxide production disrupts neuronal iron homeostasis, leading to accumulation of lipid hydroperoxides and ferroptosis-like neurotoxicity [48]. Pharmacological strategies in spinal cord injury highlight that peroxisome proliferator-activated receptor gamma (PPARγ) agonism promotes M2 polarization and tissue repair. S1P receptor modulation can shift microglia toward an M2 state via signal transducer and activator of transcription 3 (STAT3) [43,49]. As summarized in Table 1, these diverse receptors empower microglia to dynamically detect neuronal signals, orchestrate synaptic remodeling, and profoundly influence neuronal survival and circuit function across physiological and pathological states.

### 3.3. Metabolic Regulation of Microglia–Neuron Crosstalk

Microglial metabolic states act as an intrinsic switch governing neuronal plasticity and synaptic integrity. Glucose metabolism is tightly linked to microglial inflammatory responses. In pro-inflammatory states, microglia shift their primary energy source to aerobic glycolysis. However, activation of microglial cannabinoid receptor 2 (CB2) suppresses glycolytic enzymes and lactate accumulation, thereby reducing TNF-α, IL-1β, and IL-6 levels in the paraventricular nucleus (PVN) [59]. Impairments in the metabolic process disrupt essential processes like developmental synaptic pruning [60]. Conversely, enhancing metabolic flexibility, for instance via the IL-33/ST2/AKT axis, can promote such phagocytic activity [61]. These findings demonstrate that energy metabolism is fundamentally linked to microglial executive functions. Furthermore, pathological energy depletion impairs microglial motility, phagocytosis, and debris clearance, ultimately compromising neuronal immune surveillance [15]. Beyond glucose metabolism, microglial lipid homeostasis directly affects neuronal activity and development. TREM2-dependent lipid sensing and myelin phagocytosis are crucial for neuronal metabolic health, as TREM2 deficiency disrupts excitatory neuron metabolism and mitochondrial gene transcription [62]. Furthermore, TREM2 deficiency triggers cholesterol ester accumulation and LD formation in microglia, driving oxidative stress, inflammation, and a neurotoxic microenvironment [63]. Lipid metabolic homeostasis in microglia-including sensing, synthesis, and oxidation is equally essential to neuronal activity. Different fatty acids secreted by microglia differentially regulate inflammatory phenotypes: oleate promotes anti-inflammatory states, whereas palmitate drives pro-inflammatory responses, ultimately affecting synaptic pruning and neuronal connectivity [64]. In vitro studies show that iPSC-derived microglia promote brain organoid maturation via cholesterol transfer [65]. Finally, microglia can indirectly impair neurons by inducing astrocytic dysfunction. For example, Aβ-activated microglia induce reactive astrocytes to increase glycolysis and L-lactate secretion. This leads to excessive neuronal uptake via monocarboxylate transporters (MCTs), ultimately resulting in functional impairment [66]. These findings underscore the central role of bioenergetics in microglial function and offer new insights into dysregulated microglia–neuron crosstalk.

In summary, microglia dynamically regulate their functional phenotypes through energy metabolism (glycolysis/OXPHOS switching) and lipid metabolism (fatty acid sensing and lipid accumulation), thereby directly influencing synaptic pruning, neurotransmitter balance, and neuronal plasticity.

### 3.4. Direct Membrane–Membrane Contacts

**Figure 3:**
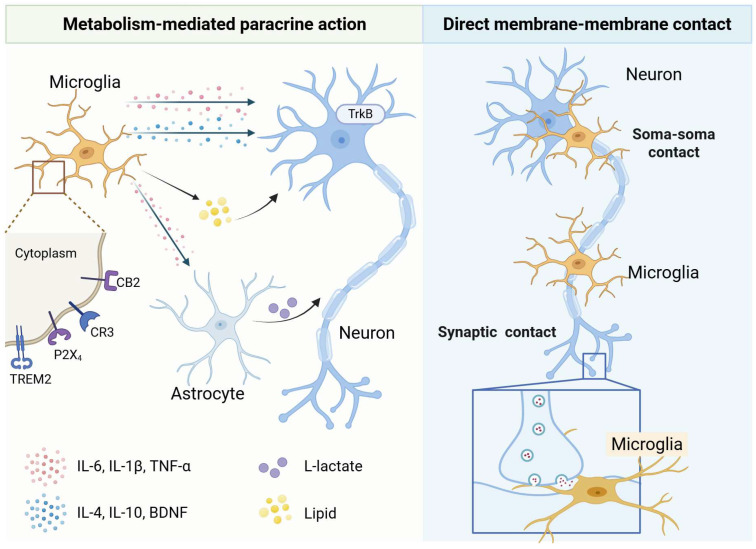
Microglial Regulation of Neurons: From Chemical Signals to Physical Contacts.

Microglia regulate neuronal activity through direct physical contacts. Bakina et al. termed a subset of microglia “satellite microglia,” which forms non-specific, tight soma-to-soma contacts with neuronal cell bodies, providing a structural basis for intercellular communication [67]. In prion-induced neurodegenerative diseases, reactive microglia establish soma-to-soma contacts with neurons a phenomenon known as neuronal encircling or enveloping that is independent of phagocytic pathways [68]. To protect against prion neurotoxicity, reactive microglia extend their cell bodies to partially envelop neuronal somata. These intimate contacts, accompanied by enhanced microglia–neuron interactions, help sustain cortical neuron viability during disease progression [68]. Additionally, Haruwaka et al. revealed that, under anesthesia, microglial processes rapidly invade the synaptic cleft to physically block GABA release onto postsynaptic membranes, thereby transiently enhancing neuronal excitability (Figure 3) [69].

## 4. Mechanisms of Neuronal Influence on Microglia

### 4.1. Chemical Regulation of Microglia by Neurons

Neurons chemically regulate microglia through neurotransmitters and other signaling molecules. In the healthy brain, dynamic microglia–neuron interactions are tightly modulated by neuronal activity [70]. For example, ATP/ADP released during neuronal activity is detected by microglia-specific P2Y_12_ receptors, triggering chemotaxis and morphological remodeling [56]. GABA and glutamate released by neurons also modulate microglial function via cognate receptors. Notably, GABA, the principal inhibitory neurotransmitter, directly suppresses microglial signaling and dampens immune responses [71]. Dopamine and serotonin receptors similarly exert complex effects. Dopamine promotes inflammatory cytokine release via dopamine receptor D3 (DRD3), whereas serotonin modulates microglial viability and NO production through HTR1A; HTR1A deletion exacerbates inflammation [72]. Neurons also engage in transmembrane “don’t-eat-me” signaling with microglia via ligand–receptor pairs. The surface receptors expressed by neurons and their corresponding ligands are summarized in Table 2. Hippocampal neurons express membrane-bound CD200, which binds microglial CD200 receptor (CD200R) to restrain activation and suppress pro-inflammatory cytokine secretion [73]. Using multi-omics approaches, Bilbo and colleagues demonstrated that excitatory neuron-derived IL-34 is essential for microglial development in the mouse anterior cingulate cortex (ACC). IL-34 deficiency reduces microglial numbers, downregulates the maturation marker transmembrane protein 119 (TMEM119), and paradoxically increases synaptic engulfment [57]. The same group previously reported that postsynaptic density protein 93 (PSD-93) drives microglial polarization by binding amino acids 357–395 of the C-X3-C motif chemokine ligand 1 (CX3CL1) chemokine ligand [74].

### 4.2. Neuronal Metabolic Homeostasis as a Master Regulator of Microglial Activity

Neurons, particularly the functional integrity of energy metabolism, are key to suppressing excessive neuroinflammation. Studies show that enhancing neuronal mitochondrial function, such as overexpressing the fusion protein Mfn2 or activating the transcriptional coactivator peroxisome proliferator-activated receptor gamma coactivator 1-alpha (PGC-1α), can effectively inhibit the pro-inflammatory activation of microglia, reducing the production of ROS and inflammatory factors, thus providing neuroprotection [83,84]. This reveals that robust neuronal metabolism is an important upstream signal curbing harmful microglial activation. Dysregulated lipid metabolism is a major driver of cellular senescence and neurodegenerative diseases. Under aging or pathological conditions, neurons, due to factors like decreased AMPK activity, transfer their accumulated lipids to surrounding microglia. These exogenous lipids form lipid droplets (LDs) within microglia, impairing their normal phagocytic clearance capacity and promoting a shift toward a pro-inflammatory phenotype [85].

### 4.3. Direct Physical Neuron-to-Microglia Contacts

**Figure 4:**
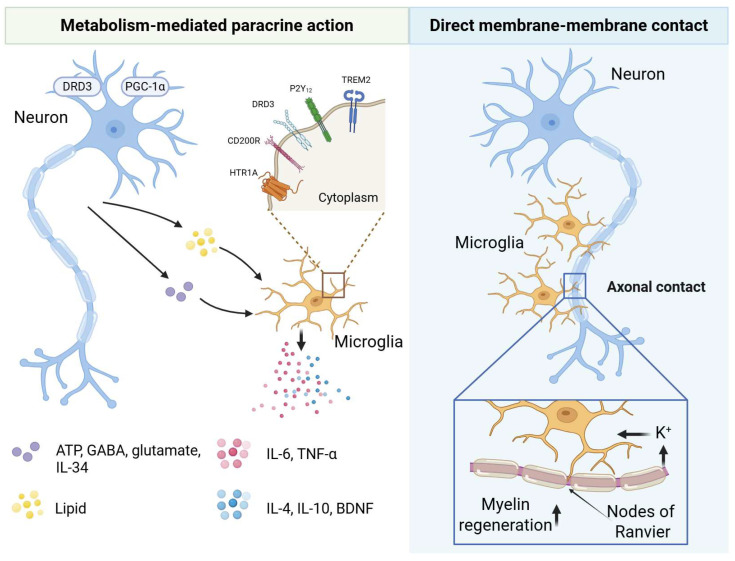
Neuron-to-Microglia Signaling: Intercellular Signal Transmission and Somatic Contacts.

Neurons reshape microglial morphology and function through direct physical contacts. Live imaging has revealed that nodes of Ranvier serve as privileged sites of direct physical interaction between microglia and neuronal axons in both mouse and human CNS. Neuronal activity-dependent potassium release modulates microglial contact with myelin and nodes of Ranvier, thereby regulating myelin remodeling and regeneration (Figure 4) [86].

## 5. Reciprocal Regulation Between Microglia and Neurons

### 5.1. Multimodal Direct Contact Networks Between Neurons and Microglia

Neurons and microglia establish a sophisticated “dialogue network” through multiple direct contact mechanisms. Functional tunneling nanotubes (TNTs) connect human microglia and neurons. These links enable the bidirectional transfer of organelles, vesicles, proteins, and pathogens, thereby generating complex intercellular networks [87]. Prion-like tau aggregates in neurons are transferred to microglia via TNTs. In return, microglia selectively deliver healthy mitochondria to aggregate-burdened neurons as a potential rescue mechanism. This bidirectional exchange is driven by neuronal ATP/ADP release and P2Y_12_ signaling, which activates the P2Y_12_R/Rac/PAK/F-actin pathway in microglia to promote TNT formation and mitigate neuronal proteotoxic stress (Table 2) [75,88]. Furthermore, the presence of α-synuclein (α-syn) aggregates also increases the number of TNT connections. Bidirectional transfer of α-syn has been detected within TNTs formed between neurons and microglia [88]. Furthermore, TNTs have been identified within human dorsal root ganglia (DRG). Regulated by MYO10, these structures mediate mitochondrial transfer from satellite glial cells (SGCs) to neighboring sensory neurons—a mechanism directly implicated in the development of diabetic peripheral neuropathy [89].

In addition, microglia establish direct physical contact with neuronal somata or processes. In both rodent and human brains, specialized somatic microglia–neuron junctions (SMNJs) form between microglial processes and neuronal cell bodies. These highly structured contacts are optimized for purinergic signaling, enabling rapid and localized communication [90]. Under homeostatic conditions, microglia establish dynamic, transient contact with neuronal somata via somatic purinergic junctions (SPJs). Microglial processes continuously extend and retract to probe neuronal health through purinergic signaling, allowing rapid responses to changes in neuronal activity [55]. Neuronal activity bidirectionally regulates the balance between microglial soma contacts and myelin phagocytosis in the optic tectum. Increased neuronal firing recruits microglia to neuronal cell bodies, reducing myelin engulfment, whereas neuronal silencing shifts microglial attention toward axons and enhances myelin pruning. This switch is governed by neuronal activity-dependent calcium transients in microglia during microglial–myelin contacts [91]. In summary, through long-range “rescue” TNTs and short-range “health patrolling” somatic junctions, neurons and microglia orchestrate a multilayered, multimodal dialogue network to collectively maintain brain homeostasis.

### 5.2. Ligand–Receptor-Mediated Bidirectional Chemical Communication

Neurons and microglia engage in bidirectional chemical communication through diverse ligand–receptor pairs (Table 2). Complement-dependent microglia–neuron signaling, which requires precise synaptic coupling, orchestrates brain development, maturation, and plasticity in both health and disease. Activated complement components—particularly cleaved protein fragments—serve as key ligands that bind cognate receptors to elicit complex biological responses [92].

Complement protein complement component 1q (C1q), secreted primarily by microglia, serves as a key initiator of complement-mediated synaptic pruning during development and disease. By tagging excess synapses, C1q facilitates their elimination and regulates neuronal migration [82]. Conversely, neurons actively internalize microglia-derived C1q in an age-dependent manner. This internalized C1q is incorporated into neuronal ribonucleoprotein (RNP) complexes, directly modulating neuronal protein translation and cellular homeostasis [93].

The CX3CL1/C-X3-C motif chemokine receptor 1 (CX3CR1) axis exemplifies the dynamic and bidirectional regulation between neurons and microglia, with its functional outcome shifting critically with physiological context. Under homeostatic conditions, neurons constitutively express the chemokine CX3CL1. This serves as a continuous “off” signal by binding to its sole receptor, CX3CR1, on microglia, actively maintaining them in a quiescent state and suppressing pro-inflammatory cytokine release [94,95,96]. Conversely, under acute inflammation, activated microglia upregulate their expression of CX3CR1 via TLR4/NF-κB signaling. This shift transforms the axis, now driving excessive synaptic engulfment and contributing to circuit dysfunction—a microglia-to-neuron regulatory effect [97]. Therefore, the CX3CL1/CX3CR1 pathway highlights the same molecular dialogue can maintain homeostasis or drive pathology, depending on which cell type initiates the signal and the state of the cellular environment.

The most dynamic example is the purinergic feedback loop centered on neuronal activity. Neurons and astrocytes release ATP during activity. Microglia sense this ATP primarily via P2Y_12_, which allows microglia to probe neuronal status. In pathology, P2Y_12_ activation can enhance pro-inflammatory cytokine release from microglia, contributing to neuronal hyperexcitability in pain models [98,99]. Through the ectonucleotidases CD39 and CD73 on surface, microglia convert ATP to adenosine. This microglia-processed adenosine then feeds back onto neurons by activating adenosine A1 receptors (A1R), powerfully suppressing further neuronal activity [100]. This establishes a precise, activity-dependent negative feedback loop where microglial sensory and metabolic activity directly modulate neuronal output.

### 5.3. Bidirectional Regulation via Cytokines and Neurotransmitters

Cytokines and neurotransmitters synergize in microglia–neuron crosstalk. Neuronal calcium transients shape microglial inflammatory states, while microglia monitor neuronal activity and release cytokines that reciprocally modulate neuronal excitability and synaptic plasticity [101,102]. In developing somatosensory cortex (P8-P10), neuronal ATP release recruit microglial processes to dendritic shafts. This contact triggers localized calcium transients and actin accumulation in microglia, driving formation of new dendritic filopodia (synaptic precursors) [103]. Microglial motility is governed by local neuronal activity (e.g., glutamate release). Through direct interactions with dendritic spines, microglia dynamically regulate synaptic stability, elimination, and formation in the adult brain [104]. Synaptic remodeling is essential for memory storage. Experience-dependent IL-33 expression in hippocampal neurons activates microglial IL-33 receptors, stimulating extracellular matrix (ECM) phagocytosis and promoting new dendritic spine formation to consolidate memory. The authors propose that IL-33 marks a synapse-strengthening neuronal subpopulation [105]. Microglia–neuron interactions also exhibit spatial heterogeneity. Integration of sc/snRNA-seq data with CellPhone DB revealed cortical microglial subpopulations that are either responsive or insensitive to specific projection neuron (PN) subtypes—likely orchestrated by niche-specific neuronal cues [106]. Neuronally released ATP activates microglial P2X_7_ receptors, which elicits TNF-α release. This TNF-α–dependent signaling promotes the clustering of GABA_A_ receptors at cortical inhibitory synapses, thereby regulating slow-wave sleep and supporting memory consolidation. [107].

### 5.4. Neuron-to-Microglia Signaling via Mitochondria

Mitochondrial transfer between microglia and neurons is a context-dependent process with dual outcomes. In supportive roles, microglia can donate healthy mitochondria to neurons. This is facilitated by mechanisms such as zinc-induced, SIRT3-dependent upregulation of Mfn2, which improves neuronal survival after spinal cord injury [108]. Donated healthy mitochondria also mitigate oxidative stress and restore normal gene expression in neurons burdened with α-syn aggregates [75]. Conversely, the transfer of damaged mitochondria is detrimental. In models like transient middle cerebral artery occlusion (tMCAO), microglia-derived dysfunctional mitochondria reduce neuronal ATP levels, exacerbate oxidative stress, and aggravate injury [109]. This bidirectional pathway is not one-sided; neurons also signal to microglia via damaged mitochondria and misfolded proteins, actively igniting microglial inflammatory activation and positioning neurons as key orchestrators of neuroinflammation [110]. Overall, mitochondrial transfer can either aggravate neurodegeneration or provide neuroprotection, highlighting its complex role [88,109,111].

### 5.5. Extracellular Vesicle-Mediated Neuron–Microglia Signaling

Extracellular vesicles (EVs), especially exosomes, are critical carriers for bidirectional signaling. Microglia-derived EVs modulate neuronal activity over long distances. Their cargo reflects microglial functional states and can influence neuronal calcium responses, participating in synaptic remodeling and pathologies like stroke and inflammation [112]. Specific harmful cargoes include cytosolic DNA and chromatin fragments from microglia with persistent DNA damage; when packaged into EVs and sent to interferon-responsive neurons, they trigger neuronal death [113]. Furthermore, activated microglia can pack the sialidase Neu3 into EVs, which disrupts neuronal network integrity upon delivery [47].

Conversely, neuron-derived EVs exert potent, context-dependent control over microglia. Under physiological conditions, they typically send inhibitory signals that promote microglial survival, suppress activation markers, and shift cytokine secretion toward an anti-inflammatory profile (e.g., increasing IL-10) [114,115]. Under stress, however, the signals change. For instance, EVs from ischemia-stressed neurons are highly enriched in miR-100-5p, which activates TLR7 in microglia via a specific motif, triggering NF-κB signaling and pro-inflammatory responses [115]. Similarly, neuron-derived exosomes can deliver miR-9-5p to microglia, suppressing SOCS2 and activating JAK/STAT3 signaling to drive pro-inflammatory M1 polarization, ultimately exacerbating neuronal damage [116,117]. Thus, neuron-derived EVs serve as versatile carriers that can reprogram microglial inflammatory states based on contextual cues.

### 5.6. Adaptive Metabolic-Immune Crosstalk Between Neurons and Microglia

**Figure 5:**
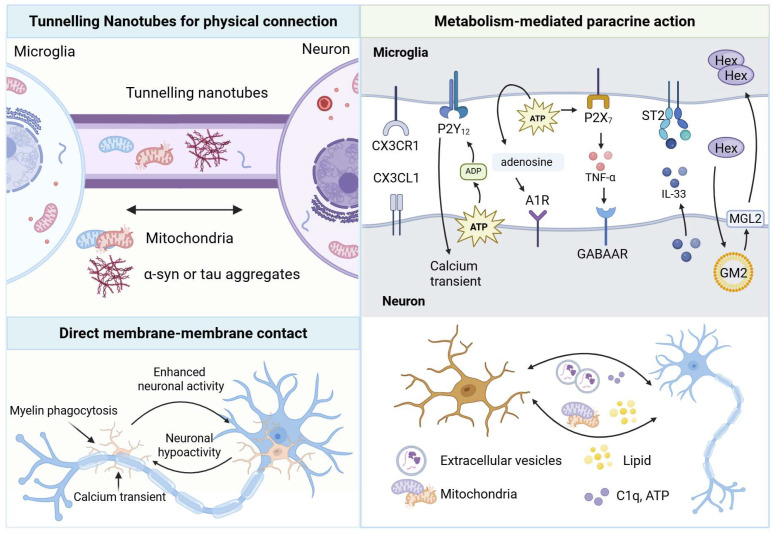
Reciprocal regulation between microglia and neurons.

A dynamic and synergistic metabolic-immune dialogue exists between neurons and microglia. Through mutual adaptation and feedback of metabolic signals, both parties collectively respond to physiological fluctuations and pathological challenges to maintain brain stability and resilience. Pro-inflammatory microglia undergo transcriptional reprogramming characterized by increased NO release and a transient rise in the local lactate-to-glucose ratio. Dynamic cerebral blood flow redistributes uneven energy substrates and oxygen to neurons, which in turn adapt their metabolism to mitigate neuroinflammatory damage [118]. Moreover, neuronal activity and health dictate microglial metabolic adaptation. When neurons become dysfunctional due to glucose deprivation, microglia exhibit metabolic flexibility by switching from glycolysis to glutaminolysis in an mTOR-dependent manner to sustain their immune surveillance role [119]. A quintessential example of mutual metabolic support is the continuous secretion of β-hexosaminidase (HexB) by homeostatic microglia. Neurons uptake HexB to degrade intracellular GM2 gangliosides, a process vital for maintaining neuronal lipid homeostasis and health [120]. The breakdown of this dialogue, as seen in lysosomal storage disorders where HexB secretion is lost, leads to toxic lipid accumulation in neurons, triggering neuroinflammation and cell death. These findings highlight a sophisticated metabolic-immune dialogue that serves as the cornerstone of neuronal and microglial resilience to physiological and pathological challenges (Figure 5).

## 6. Microglia–Neuron Crosstalk in Neurodegenerative Diseases

### 6.1. Alzheimer’s Disease: Biphasic and Spatiotemporally Dynamic Microglia–Neuron Crosstalk

Alzheimer’s disease (AD) is a progressive neurodegenerative disorder of the CNS. It initially presents as mild cognitive impairment and is characterized by cerebral β-amyloid (Aβ) deposition, accumulation of hyperphosphorylated tau, and synaptic loss, with relentless clinical worsening. Throughout disease progression, microglia and neurons engage in intense bidirectional crosstalk via multiple signaling axes. This interaction exhibits a striking biphasic nature across different disease stages. In early AD, microglia cluster around Aβ plaques through TREM2–apolipoprotein E (APOE)-dependent activation of the DAM program. They form a compact barrier that restricts diffusion of toxic oligomers and shields dystrophic neurites from oxidative stress propagation [50]. With disease progression, however, microglia lose homeostatic signatures, adopt a chronic pro-inflammatory phenotype, and undergo barrier collapse, inflammatory amplification, and direct neurotoxicity. This spatiotemporal heterogeneity confers on microglia a dual identity: protective guardians in early disease and detrimental accomplices in late stages.

Ligand–receptor interactions between microglia and neurons drive the pathogenesis of AD. Hyperphosphorylated tau competes with neuronal CX3CL1 for binding to microglial CX3CR1, facilitating uptake. However, microglia internalize hyperphosphorylated tau far less efficiently, uncoupling activation from phagocytosis, chronically disrupting the CX3CL1/CX3CR1 axis, and accelerating tau propagation [53]. Concomitantly, aberrant early activation of the classical complement cascade (C1q–C3–CR3) drives excessive synaptic engulfment—a primary mechanism of synapse loss in AD. Inhibition of C1q, C3, or CR3 markedly attenuates early synaptic pathology [121]. Neurons actively counter this process via “don’t-eat-me” signaling: CD47 overexpression reverts microglia from DAM to homeostatic phenotype and substantially reduces Aβ-induced synapse loss [122]. High-resolution imaging in AD models reveals a marked reduction in microglial contacts with interneuron dendrites and a shift toward dominant soma interactions, indicating pathological rewiring of hippocampal network control [123]. Strikingly, selective microglial depletion at mid-late stages in 10-month-old 5× FAD mice rescues dendritic spines and neurons, underscoring a complete reversal from early protection to late-stage harm [124].

Genetic risk factors modulate neuroimmunometabolic coupling between microglia and neurons. APOE4 and TREM2 variants represent the strongest genetic risk factors for late-onset AD. Microglia promote APOE4-associated Aβ and tau pathology in human neurons [125]. The APOE4 allele severely compromises microglial homeostasis and induces lipid metabolic reprogramming: increased intracellular neutral lipid storage, downregulated fatty acid receptor CD36, and aberrant upregulation of lipid synthesis genes. Consequently, neuronal surveillance and purinergic signaling are impaired [126]. Critically, excess cholesterol released by APOE4 microglia accumulates in neuronal lipid rafts, activates GIRK3 channels, hyperpolarizes resting membrane potential, suppresses neuronal excitability, and disrupts network activity [127]. In addition, TREM2 loss-of-function causes autophagic defects, massive autophagosome accumulation, and collapse of energy metabolism under both homeostatic and stressed conditions [128]. Cerebral glucose hypometabolism and mitochondrial dysfunction are among the earliest shared pathologies in AD, often preceding Aβ deposition [129]. Recent evidence shows that enhancing glycolysis and OXPHOS by targeting HKII/PDK2 (e.g., with cordycepin) reverses aberrant microglial metabolic reprogramming, shifts polarization from M1 to M2, and markedly improves neuronal survival [130]. Collectively, these findings indicate that genetic risk factors rewire the “nutritive–toxic” balance between microglia and neurons at the metabolic level.

In conclusion, microglia–neuron interactions in AD encompass three core dimensions—immune recognition or clearance, genetic-metabolic coupling, and synaptic pruning regulation. Their spatiotemporal dynamics and functional biphasicity critically dictate disease trajectory and provide a solid rationale for stage-specific, precision microglial targeting therapies (Table 3).

### 6.2. Parkinson’s Disease: Microglial–Neuronal Crosstalk in α-Synuclein Pathology

Parkinson’s disease (PD) is a multisystem neurodegenerative disorder characterized by misfolded fibrillar α-syn, progressive loss of dopaminergic neurons in the substantia nigra pars compacta (SNpc), degeneration of the nigrostriatal pathway, and Lewy body formation. α-Syn constitutes the principal component of Lewy bodies [133], and its substantial accumulation in both microglia and astrocytes is a hallmark of PD mouse models [152]. Neuron-released α-syn activates β1-integrin to recruit microglia to pathological sites while simultaneously triggering pro-inflammatory activation via Toll-like receptor 2 (TLR2) signaling [131]. Aggregated α-syn drives microglial polarization toward the pro-inflammatory M1 phenotype and induces secretion of factors (IL-1α, TNF-α, C1q) that convert astrocytes into neurotoxic A1 cells, culminating in dopaminergic neuron death [132]. These findings underscore the central role of glia in amplifying α-syn neurotoxicity.

Microglial metabolic state directly governs α-syn handling capacity and determines whether microglia protect or damage dopaminergic neurons. Capsaicin, acting via transient receptor potential vanilloid 1 (TRPV1), restores energy metabolism and phagocytic activity in PD cellular models by engaging the Ca^2+^/CaMKK2/AMPK/mTOR pathway. This reduces phosphorylated α-syn propagation and aggregation in neurons while enhancing its microglial uptake and degradation [133,134]. Conversely, suppressing excessive microglial glycolysis ameliorates neuroinflammation and protects dopaminergic neurons in vivo [153]. Clinically, terazosin—an approved PGK1 activator that boosts glycolysis and ATP production—has been repurposed for PD and is associated with reduced incidence and slower progression [135]. Inflammation and energy metabolism pathways are reciprocally regulated. IL-4-induced anti-inflammatory microglia prevent dopaminergic neuron degeneration and neuroinflammation in PD mice by suppressing deleterious ARRB1 while enhancing protective ARRB2, thereby restoring mitochondrial function via the Samd4/mTOR/OXPHOS axis and establishing a neuron-friendly microglial phenotype [136]. Together, these data establish precise modulation of the microglial metabolism–inflammation axis as one of the most promising therapeutic strategies in PD.

Direct intercellular transfer pathways drive α-syn propagation in PD. Beyond classical secretion, two novel direct transfer mechanisms are critical for α-syn spread. Exosomes are major vehicles of α-syn aggregate transmission in PD. Microglia-derived exosomes transfer α-syn aggregates to neighboring neurons; these exosomes are markedly elevated in PD patient CSF and induce neuronal protein aggregation [154]. α-Syn rapidly induces energy failure at striatal dopaminergic axon terminals and accumulation of dysfunctional mitochondria [152,155]. In PD models (Table 3), TNTs form dynamic physical bridges between neurons and microglia, enabling bidirectional transfer of α-syn and mitochondria. Microglia degrade neuron-derived pathological α-syn while delivering healthy mitochondria to energy-depleted dopaminergic terminals—a dual role of clearance and acute metabolic rescue within the same conduit [88]. These direct and indirect physical conduits provide novel mechanisms for relay-like α-syn propagation and open new avenues for precision interventions to block pathological protein spread (Table 3).

### 6.3. Multiple Sclerosis: Central and Biphasic Role of Microglia

Multiple sclerosis (MS) is a chronic inflammatory neurodegenerative disease characterized by focal or diffuse demyelination, axonal exposure, and progressive neuronal loss. Under physiological conditions, lipid-rich myelin ensures rapid saltatory conduction of action potentials and provides essential metabolic support to axons via lactate shuttling. Upon lesion formation, activated microglia-together with peripherally derived monocytes/macrophages, T lymphocytes (particularly Th17 cells), and B lymphocytes-rapidly infiltrate the site, establishing a prototypical inflammatory microenvironment [138,156].Notably, microglia play a central, stage-dependent role throughout the progression of MS (Table 3).

Surface receptors on microglia and neurons sense cytokines to mediate stage-specific interactions in MS. In the acute phase, TREM2-dependent phagocytosis clears myelin debris and infiltrating Th17 cells. As a key receptor for phagocytosis and lipid sensing, upregulated TREM2 markedly increases oligodendrocyte precursor cell (OPC) density in demyelinated regions, promoting mature oligodendrocyte differentiation and remyelination. This indirectly preserves axonal integrity and neuronal function while facilitating tissue repair [31,139]. Concurrently, neuronal soluble CX3CL1 binds microglial CX3CR1 to restrain excessive pro-inflammatory polarization and sustain secretion of neurotrophic factors such as BDNF and IGF-1. In progressive stages, however, elevated receptor-interacting protein kinase 1 (RIPK1) in microglia and astrocytes drives necroptosis and perpetuates a pro-inflammatory milieu. Released mediators such as TNF-α, IL-1β, and ROS directly induce axonal energy crisis and neuronal apoptosis while sustaining smoldering inflammation, thereby driving transition from relapsing-remitting to secondary or progressive MS [157,158]. Additionally, myelin-derived oxidized phosphatidylcholines (OxPCs)—potent markers of oxidative stress—synergize with debris to activate microglial TLR4/NF-κB signaling. This amplifies diffusion of TNF-α and IL-1β toward neurons, causing excitotoxicity and synaptic loss [140]. These alterations exacerbate pathology in both experimental autoimmune encephalomyelitis (EAE) and human progressive MS [159]. These dynamic mechanisms underscore the pivotal role of microglia–neuron communication throughout the MS disease course.

Microglial–neuronal metabolic coupling in MS shifts from supportive to complicit. Single-cell transcriptomics reveals that lesion-associated DAM upregulate genes involved in iron metabolism and lipid peroxidation. This leads to the emergence of iron-laden and foamy microglia, which amplify neurotoxicity and correlate with cognitive impairment [141]. Activated microglia exhibit excessive glycolysis and produce abundant ROS. These impair neighboring axonal complex I via reverse electron transport, resulting in “virtual hypoxia” and ATP depletion. In chronic lesions, abnormal accumulation of iron and lipid droplets in microglia (iron-laden and foamy phenotypes) releases lipotoxic metabolites, disrupting the neuronal tricarboxylic acid (TCA) cycle and OXPHOS [143]. Microglial activation is not confined to lesions but extends into normal-appearing white matter (NAWM). Although MS arises from broader immune dysregulation, this pattern implicates microglia as early drivers of progressive axonal degeneration. Single-nucleus RNA sequencing (snRNA-seq) confirms lineage- and region-specific transcriptional reprogramming in microglia, astrocytes, and oligodendrocytes across cortical and white matter regions in MS models. This collectively exacerbates gray matter synaptic stripping and neuronal atrophy [144]. Emerging evidence shows markedly reduced bile acid metabolism in MS patients. Microglia can internalize endogenous tauroursodeoxycholic acid (TUDCA) via G-protein-coupled bile acid receptor 1 (GPBAR1), effectively preventing polarization toward neurotoxic phenotypes and indicating potential neuroprotective effects of bile acid signaling [142]. Targeting central myeloid cell infiltration or restoring metabolic balance has shown robust neuroprotection in progressive MS models, providing strong preclinical support for clinical translation [160].

## 7. Future Challenges

Neurons, as high-energy consumers, influence nearby microglia through the release of metabolites, signaling lipids, and mitochondrial components, but the mechanisms governing this exchange remain fragmented. While pathways like the neuronal lactate shuttle are recognized [66], the full repertoire of exchanged metabolites and their spatiotemporal dynamics in vivo are poorly mapped. Similarly, the signaling logic of lipid transfer—particularly how neuron-derived lipids are sensed by receptors like TREM2 to modulate microglial function—needs to be elucidated [126]. Moreover, although tunneling nanotubes facilitate mitochondrial transfer [87,88], the molecular triggers that determine whether transferred mitochondria are salutary or deleterious are unclear.

Systematic understanding remains limited regarding human microglial heterogeneity, the functions of disease-associated subsets across brain regions and microenvironments, and the precise mechanisms by which neurons regulate microglia through metabolic coupling. This knowledge gap is particularly evident when contrasting insights from rodent models with the emerging complexity of the human brain [161]. For instance, while animal studies have identified microglial subtypes such as the DAM in AD models, their precise human equivalents and regional specializations are still being mapped [162]. Recent single-nucleus RNA sequencing of post-mortem human brain tissue has revealed a strikingly diverse microglial landscape, with distinct transcriptional signatures in cortical versus white matter regions, and further nuances within the hippocampus, amygdala, and substantia nigra [163]. In cortical regions including the prefrontal cortex, microglia from individuals with major depressive disorder display a distinct, largely non-inflammatory transcriptional state with altered expression of immune effector and homeostatic genes, which differs from the activated, phagocytic phenotypes typically induced by chronic stress in rodent models [164,165,166,167]. Hyper-ramified microglial morphologies have been linked to depressive-like behavior in stress paradigms, and type I interferon signaling is known to impose a unique gene signature and morphological state on microglia, suggesting a disease-relevant, interferon-skewed microglial program that current chronic stress models only partly reproduce [168,169,170]. Conversely, in PD, a specific microglial subset characterized by elevated GPNMB expression is enriched in the substantia nigra [171,172], where it appears to engage in dysfunctional interactions with dopaminergic neurons, possibly exacerbating neuronal loss through impaired phagocytosis of α-syn aggregates [173,174].

Bridging the translational gap between rodent models and human neurodegeneration is essential, as human microglia differ markedly from mouse microglia in transcriptional programs, aging trajectories, and neurodegeneration-related modules despite sharing core markers such as P2Y_12_ [175,176,177]. In Aβ mouse models, microglia typically follow a relatively uniform DAM trajectory, whereas human microglia in AD display a broader continuum of DAM-like, interferon, and MHC-II/HLA antigen-presentation states that only partially overlap with mouse DAM and are strongly modulated by APOE genotype [20,178,179]. Advanced human-based systems, including iPSC-derived microglia xenografted into mouse brain and microglia-containing cerebral organoids, now enable dynamic study of these human-specific microglial states and their interactions with Aβ and neural cells in more physiological contexts [65,172,180,181].

## 8. Concluding Remarks

Microglia–neuron crosstalk is essential for maintaining brain homeostasis and regulating circuit function. In neurodegenerative diseases such as AD, PD, and MS, this interaction exhibits striking spatiotemporal dynamics. Extensive evidence shows that microglia transition from homeostatic guardians to drivers of inflammatory amplification across disease stages. This shift is accompanied by profound transcriptomic remodeling and metabolic reprogramming, including enhanced glycolysis, mitochondrial failure, and dysregulated lipid metabolism. Concurrently, neurons continuously shape microglial states through synaptic activity, metabolic cues, and transcellular transfer, conferring marked cell-type and niche-specific phenotypes under pathological conditions. To translate this knowledge into therapies, future research must bridge multiple scales of investigation. Success in this endeavor will depend on defining the causal logic of microglia–neuron communication across disease stages, thereby revealing actionable targets within the immune–metabolic network for precision intervention.

## Figures and Tables

**Table 1:** Surface receptors and binding ligands of microglia.

Microglial Receptor Category	Representative Receptors	Ligands Bound	Ligands Source	Primary Functions	Reference
**Pattern** **Recognition** **Receptors**	Toll-like Receptors	PAMPs, DAMPs	Pathogens, damaged/dead cells	Innate immune surveillance Pathogen/damage recognition Neuroinflammatory response initiation.	[25]
**Immunoglobulin** **Superfamily** **Receptors**	TREM2	Lipids, ApoE, Aβ, apoptotic debris	Neurons, apoptotic cells	Phagocytosis regulation Anti-inflammatory control Cell survival promotion	[50]
	RAGE	AGEs, Aβ, HMGB1	Injured or stressed cells	Chronic inflammation mediation Damage exacerbation	[51]
	CD200R	CD200	Neurons, reactive astrocytes	Microglial activation inhibition CNS immune homeostasis maintenance	[52]
**Chemokine** **Receptors**	CX3CR1	CX3CL1	Neurons (membrane-bound/secreted)	Microglial surveillance maintenance Neuron–microglia communication Synaptic pruning regulation	[53]
	CCR2	CCL2	Activated glia or neurons	Peripheral monocyte recruitment to CNS Microglia/macrophage accumulation at inflammatory sites	[54]
**Neurotransmitter or** **Purinergic** **Receptors**	P2Y_12_	ADP	Neurons (active/damaged)	Chemotactic response to neuronal activity Guidance toward injury/activity sites	[55,56]
**Cytokine or Growth Factor Receptors**	CSF1R	CSF1, IL-34	Neurons (secreted)	Critical for microglial survival/proliferation Homeostatic function maintenance	[57]
	TNFR/IL-1R	TNF-α, IL-1β	Activated glia/immune cells	Inflammatory signal reception Neuroimmune response amplification/modulation	[58]

**Table 2:** Surface receptors and binding ligands of neurons.

Neuronal Receptor Category	Representative Receptors	Ligands Bound	Ligands Source	Primary Functions	Reference
**Ionotropic Neurotransmitter** **Receptors**	NMDARs AMPARs KARs	Glutamate	Excitatory neurons	Fast excitatory synaptic transmission NMDARs: key molecular switches for LTP Learning and memory facilitation	[71]
	GABAₐRs GlyRs	GABA, Glycine	Inhibitory neurons	Fast inhibitory synaptic transmission Reduction in neuronal excitability Circuit balance maintenance	
	P2X_4_R	ATP	Purinergic neurons, glia	Fast purinergic signaling Pain transmission Inflammatory response participation	[75]
**Metabotropic Neurotransmitter** **Receptors**	mGluRs	Glutamate	Excitatory neurons	G-protein coupled signaling Synaptic plasticity modulation Neuronal excitability regulation Neurotransmitter release control	[62]
	mAChRs	Acetylcholine	Cholinergic neurons	Slow neuromodulation by ACh Influence on learning, memory, and attention	[76]
	DARs 5-HTRs	Dopamine, Serotonin, etc.	Monoaminergic neurons	Widespread slow modulation by monoamines Higher-order function regulation (mood, reward, motor control)	[72]
**Neuropeptide and** **Neuromodulator Receptors**	μORs	Endorphins, Enkephalins, etc.	Neurons	Pain perception modulation Reward and emotion regulation Analgesia and euphoria production	[77]
	NKRs	Substance P, etc.	Neurons	Pain transmission Neurogenic inflammation participation	[78]
**Neurotrophic Factor** **Receptors**	Trk Receptor Family	NGF, BDNF, NT-3/4	Target cells, glia	Neuronal survival, growth, differentiation Synaptic plasticity regulation Long-term functional modulation	[79]
	p75ᴺᵀᴿ	Various neurotrophic factors	Neurons, glia	Dual function: cell survival or apoptosis Complex co-receptor for Trk receptors	[80]
**Immune and Cytokine** **Receptors**	TNF Receptors, IL-1 Receptors	TNF-α, IL-1β	Microglia, astrocytes	Neuro-immune crosstalk mediation Pathological overactivation leading to inflammatory damage and neuronal death	[81,82]
	CRs	C1q, C3b, etc.	Microglia	Synapse tagging by complement system Facilitation of synaptic pruning and elimination (development and disease)	

**Table 3:** The role of the microglia–neurons crosstalk in driving the progression of the different neurodegenerations.

Disease	Contact-Dependent Signaling	Soluble Mediators	Metabolic Coupling	Synaptic Remodeling
Alzheimer’s Disease	CX3CL1/CX3CR1 disrupted by p-tau [53]	Early complement (C1q–C3–CR3) drives synaptic loss [121] CD47 “don’t-eat-me” signal [122]	APOE4 disrupts lipid metabolism [126,127] TREM2 loss [128] Glycolysis/OXPHOS enhancement protective [130]	Complement-mediated pruning [121] Late microglial depletion rescues spines [124]
Parkinson’s Disease	α-Syn activates via TLR2 & β1-integrin [131]	α-Syn polarizes microglia, secreting IL-1α, TNF-α, C1q [132]	TRPV1 activation restores phagocytosis via Ca^2+^/AMPK/mTOR [133,134] PGK1 activator (terazosin) slows progression [135] IL-4 restores mitochondria via Samd4/mTOR/OXPHOS [136]	Corticostriatal plasticity changes [137]
Multiple Sclerosis	TREM2 phagocytosis clears debris and promotes remyelination [31,138,139] CX3CL1/CX3CR1 anti-inflammatory [139] TLR4/NF-κB by OxPCs [140]	RIPK1 necroptosis release TNF-α, IL-1β, axonal energy crisis [133,134]	DAM disrupts neuronal TCA/OXPHOS [141,142,143] Glycolysis, axonal “virtual hypoxia” [143] TUDCA via GPBAR1 neuroprotective [142,144]	Synaptic stripping and neuronal atrophy in gray matter [139]
Huntington’s Disease	TYROBP/TREM2 module [145] TLR–MyD88–NF-κB–driven microglial activation [145,146] Cell-autonomous mHTT effects [147]	IL-1β/IL-6/IL-8/TNF-α; CCL3/4/5–CCR5 autophagy block [148] C1q/C3-mediated synapse tagging [149]	ENT3-dependent microglial metabolism [150] KMO–kynurenine pathway [146] Oxidative stress; proposed TNT mitochondrial transfer [75]	Microglia–complement-dependent pruning of corticostriatal synapses [75] ECM/perineuronal net remodeling [151]

## Data Availability

No new data were created or analyzed in this study. Data sharing is not applicable to this article.

## References

[1] Wang L., Liu T., Wang X., Tong L., Chen G., Zhou S., Zhang H., Liu H., Lu W., Wang G. (2023). Microglia-derived TNF-alpha contributes to RVLM neuronal mitochondrial dysfunction via blocking the AMPK-Sirt3 pathway in stress-induced hypertension. J. Neuroinflammation.

[2] Gomez-Nicola D., Perry V.H. (2015). Microglial dynamics and role in the healthy and diseased brain: A paradigm of functional plasticity. Neuroscientist.

[3] Salter M.W., Beggs S. (2014). Sublime microglia: Expanding roles for the guardians of the CNS. Cell.

[4] Nayak D., Roth T.L., McGavern D.B. (2014). Microglia development and function. Annu. Rev. Immunol..

[5] Rossi F., Lewis C. (2018). Microglia’s heretical self-renewal. Nat. Neurosci..

[6] Hickman S., Izzy S., Sen P., Morsett L., El Khoury J. (2018). Microglia in neurodegeneration. Nat. Neurosci..

[7] Bi Q., Wang C., Cheng G., Chen N., Wei B., Liu X., Li L., Lu C., He J., Weng Y. (2022). Microglia-derived PDGFB promotes neuronal potassium currents to suppress basal sympathetic tonicity and limit hypertension. Immunity.

[8] Wood L.B., Singer A.C. (2025). Neurons as Immunomodulators: From Rapid Neural Activity to Prolonged Regulation of Cytokines and Microglia. Annu. Rev. Biomed. Eng..

[9] Bernier L.P., York E.M., MacVicar B.A. (2020). Immunometabolism in the Brain: How Metabolism Shapes Microglial Function. Trends Neurosci..

[10] Madore C., Yin Z., Leibowitz J., Butovsky O. (2020). Microglia, Lifestyle Stress, and Neurodegeneration. Immunity.

[11] Yang Y., Zhao X., Zhu Z., Zhang L. (2022). Vascular dementia: A microglia’s perspective. Ageing Res. Rev..

[12] Castro-Gomez S., Heneka M.T. (2024). Innate immune activation in neurodegenerative diseases. Immunity.

[13] Prinz M., Jung S., Priller J. (2019). Microglia Biology: One Century of Evolving Concepts. Cell.

[14] Munro D.A.D., Bestard-Cuche N., McQuaid C., Chagnot A., Shabestari S.K., Chadarevian J.P., Maheshwari U., Szymkowiak S., Morris K., Mohammad M. (2024). Microglia protect against age-associated brain pathologies. Neuron.

[15] Beccari S., Sierra-Torre V., Valero J., Pereira-Iglesias M., Garcia-Zaballa M., Soria F.N., De Las Heras-Garcia L., Carretero-Guillen A., Capetillo-Zarate E., Domercq M. (2023). Microglial phagocytosis dysfunction in stroke is driven by energy depletion and induction of autophagy. Autophagy.

[16] Hasavci D., Blank T. (2024). A microglial compliment: Controlling neuronal function from within. Signal Transduct. Target. Ther..

[17] Kwon H.S., Koh S.H. (2020). Neuroinflammation in neurodegenerative disorders: The roles of microglia and astrocytes. Transl. Neurodegener..

[18] Paolicelli R.C., Sierra A., Stevens B., Tremblay M.E., Aguzzi A., Ajami B., Amit I., Audinat E., Bechmann I., Bennett M. (2022). Microglia states and nomenclature: A field at its crossroads. Neuron.

[19] Ransohoff R.M. (2016). A polarizing question: Do M1 and M2 microglia exist?. Nat. Neurosci..

[20] Chen Y., Colonna M. (2021). Microglia in Alzheimer’s disease at single-cell level. Are there common patterns in humans and mice?. J. Exp. Med..

[21] Silvin A., Uderhardt S., Piot C., Da Mesquita S., Yang K., Geirsdottir L., Mulder K., Eyal D., Liu Z., Bridlance C. (2022). Dual ontogeny of disease-associated microglia and disease inflammatory macrophages in aging and neurodegeneration. Immunity.

[22] Krasemann S., Madore C., Cialic R., Baufeld C., Calcagno N., El Fatimy R., Beckers L., O’Loughlin E., Xu Y., Fanek Z. (2017). The TREM2-APOE Pathway Drives the Transcriptional Phenotype of Dysfunctional Microglia in Neurodegenerative Diseases. Immunity.

[23] Li Q., Cheng Z., Zhou L., Darmanis S., Neff N.F., Okamoto J., Gulati G., Bennett M.L., Sun L.O., Clarke L.E. (2019). Developmental Heterogeneity of Microglia and Brain Myeloid Cells Revealed by Deep Single-Cell RNA Sequencing. Neuron.

[24] Marschallinger J., Iram T., Zardeneta M., Lee S.E., Lehallier B., Haney M.S., Pluvinage J.V., Mathur V., Hahn O., Morgens D.W. (2020). Lipid-droplet-accumulating microglia represent a dysfunctional and proinflammatory state in the aging brain. Nat. Neurosci..

[25] Wang Q., Yang S., Zhang X., Zhang S., Chen L., Wang W., Chen N., Yan J. (2024). Inflammasomes in neurodegenerative diseases. Transl. Neurodegener..

[26] Zhao N., Bu G. (2023). A TREM2 antibody energizes microglia. Nat. Neurosci..

[27] Ting K.K.Y. (2024). Fructose overconsumption-induced reprogramming of microglia metabolism and function. Front. Immunol..

[28] Chen H., Guo Z., Sun Y., Dai X. (2023). The immunometabolic reprogramming of microglia in Alzheimer’s disease. Neurochem. Int..

[29] Everts B., Amiel E., Huang S.C., Smith A.M., Chang C.H., Lam W.Y., Redmann V., Freitas T.C., Blagih J., van der Windt G.J. (2014). TLR-driven early glycolytic reprogramming via the kinases TBK1-IKKvarepsilon supports the anabolic demands of dendritic cell activation. Nat. Immunol..

[30] Schuster S., Boley D., Moller P., Stark H., Kaleta C. (2015). Mathematical models for explaining the Warburg effect: A review focussed on ATP and biomass production. Biochem. Soc. Trans..

[31] Nugent A.A., Lin K., van Lengerich B., Lianoglou S., Przybyla L., Davis S.S., Llapashtica C., Wang J., Kim D.J., Xia D. (2020). TREM2 Regulates Microglial Cholesterol Metabolism upon Chronic Phagocytic Challenge. Neuron.

[32] Bartels T., De Schepper S., Hong S. (2020). Microglia modulate neurodegeneration in Alzheimer’s and Parkinson’s diseases. Science.

[33] Tsuda M., Shigemoto-Mogami Y., Koizumi S., Mizokoshi A., Kohsaka S., Salter M.W., Inoue K. (2003). P2X4 receptors induced in spinal microglia gate tactile allodynia after nerve injury. Nature.

[34] Ulmann L., Hatcher J.P., Hughes J.P., Chaumont S., Green P.J., Conquet F., Buell G.N., Reeve A.J., Chessell I.P., Rassendren F. (2008). Up-regulation of P2X4 receptors in spinal microglia after peripheral nerve injury mediates BDNF release and neuropathic pain. J. Neurosci..

[35] Zhao X., Zhang J., Qiu F., Cai C., Zhang Y., Wang H., Chen C., Lu J. (2025). Salidroside Alleviates Methamphetamine-Induced Cognitive Impairment by Targeting the AKT Pathway to Reduce Neuronal Apoptosis and Neuroinflammation. Neuropharmacol. Ther..

[36] Liddelow S.A., Guttenplan K.A., Clarke L.E., Bennett F.C., Bohlen C.J., Schirmer L., Bennett M.L., Munch A.E., Chung W.S., Peterson T.C. (2017). Neurotoxic reactive astrocytes are induced by activated microglia. Nature.

[37] Wang P., Zhang J. (2022). Persistent expression of NLRP3 in spinal microglia promotes development of lumbar disc degeneration. Front. Immunol..

[38] Lee E., Chang Y. (2025). Modulating Neuroinflammation as a Prospective Therapeutic Target in Alzheimer’s Disease. Cells.

[39] Willis E.F., MacDonald K.P.A., Nguyen Q.H., Garrido A.L., Gillespie E.R., Harley S.B.R., Bartlett P.F., Schroder W.A., Yates A.G., Anthony D.C. (2020). Repopulating Microglia Promote Brain Repair in an IL-6-Dependent Manner. Cell.

[40] Yu D., Jain S., Wangzhou A., Zhu B., Shao W., Coley-O’Rourke E.J., De Florencio S., Kim J., Choi J.J., Paredes M.F. (2025). Microglia regulate GABAergic neurogenesis in prenatal human brain through IGF1. Nature.

[41] Francos-Quijorna I., Amo-Aparicio J., Martinez-Muriana A., López-Vales R. (2016). IL-4 drives microglia and macrophages toward a phenotype conducive for tissue repair and functional recovery after spinal cord injury. Glia.

[42] Ma L., Pan X., Zhou F., Liu K., Wang L. (2018). Hyperforin protects against acute cerebral ischemic injury through inhibition of interleukin-17A-mediated microglial activation. Brain Res..

[43] Akhmetzyanova E., Kletenkov K., Mukhamedshina Y., Rizvanov A. (2019). Different Approaches to Modulation of Microglia Phenotypes After Spinal Cord Injury. Front. Syst. Neurosci..

[44] Favuzzi E., Huang S., Saldi G.A., Binan L., Ibrahim L.A., Fernandez-Otero M., Cao Y., Zeine A., Sefah A., Zheng K. (2021). GABA-receptive microglia selectively sculpt developing inhibitory circuits. Cell.

[45] Deng L., Song S.Y., Zhao W.M., Meng X.W., Liu H., Zheng Q., Peng K., Ji F.H. (2024). Triggering Receptor Expressed on Myeloid Cells 2 Alleviated Sevoflurane-Induced Developmental Neurotoxicity via Microglial Pruning of Dendritic Spines in the CA1 Region of the Hippocampus. Neurosci. Bull..

[46] Hu X., Zhang D., Pang H., Caudle W.M., Li Y., Gao H., Liu Y., Qian L., Wilson B., Di Monte D.A. (2008). Macrophage antigen complex-1 mediates reactive microgliosis and progressive dopaminergic neurodegeneration in the MPTP model of Parkinson’s disease. J. Immunol..

[47] Chen S.H., Han S., Hu C.F., Zhou R., Gao Y., Tu D., Gao H., Feng J., Wang Y., Lu R.B. (2022). Activation of the MAC1-ERK1/2-NOX2 Pathway Is Required for LPS-Induced Sustaining Reactive Microgliosis, Chronic Neuroinflammation and Neurodegeneration. Antioxidants.

[48] Wang Q., Liu J., Zhang Y., Li Z., Zhao Z., Jiang W., Zhao J., Hou L., Wang Q. (2024). Microglial CR3 promotes neuron ferroptosis via NOX2-mediated iron deposition in rotenone-induced experimental models of Parkinson’s disease. Redox Biol..

[49] Qin C., Fan W.H., Liu Q., Shang K., Murugan M., Wu L.J., Wang W., Tian D.S. (2017). Fingolimod Protects Against Ischemic White Matter Damage by Modulating Microglia Toward M2 Polarization via STAT3 Pathway. Stroke.

[50] Wendt S., Johnson S., Weilinger N.L., Groten C., Sorrentino S., Frew J., Yang L., Choi H.B., Nygaard H.B., MacVicar B.A. (2022). Simultaneous imaging of redox states in dystrophic neurites and microglia at Abeta plaques indicate lysosome accumulation not microglia correlate with increased oxidative stress. Redox Biol..

[51] Guan L., Mao Z., Yang S., Wu G., Chen Y., Yin L., Qi Y., Han L., Xu L. (2022). Dioscin alleviates Alzheimer’s disease through regulating RAGE/NOX4 mediated oxidative stress and inflammation. Biomed. Pharmacother..

[52] Zhang L., Xu J., Gao J., Wu Y., Yin M., Zhao W. (2018). CD200-, CX3CL1-, and TREM2-mediated neuron-microglia interactions and their involvements in Alzheimer’s disease. Rev. Neurosci..

[53] Bolos M., Llorens-Martin M., Perea J.R., Jurado-Arjona J., Rabano A., Hernandez F., Avila J. (2017). Absence of CX3CR1 impairs the internalization of Tau by microglia. Mol. Neurodegener..

[54] Gao F., Zhang P.F., Gao J., Song J., Chi S., Alzheimer’s Disease Neuroimaging Initiative (2022). The CCL2 rs4586 SNP Is Associated with Slower Amyloid-beta Deposition and Faster Tau Accumulation of Alzheimer’s Disease. J. Alzheimers Dis..

[55] Makarava N., Safadi T., Bocharova O., Mychko O., Pandit N.P., Molesworth K., Eyo U.B., Baskakov I.V. (2025). Knockout of P2Y12 receptor facilitates neuronal envelopment by reactive microglia and accelerates prion disease. J. Neuroinflammation.

[56] Sipe G.O., Lowery R.L., Tremblay M.E., Kelly E.A., Lamantia C.E., Majewska A.K. (2016). Microglial P2Y12 is necessary for synaptic plasticity in mouse visual cortex. Nat. Commun..

[57] Devlin B.A., Nguyen D.M., Ribeiro D., Grullon G., Clark M.J., Finn A., Ceasrine A.M., Oxendine S., Deja M., Shah A. (2025). Excitatory-neuron-derived interleukin-34 supports cortical developmental microglia function. Immunity.

[58] Valiukas Z., Tangalakis K., Apostolopoulos V., Feehan J. (2025). Microglial activation states and their implications for Alzheimer’s Disease. J. Prev. Alzheimers Dis..

[59] Shan R., Zhang Y., Shi Y., Wang X., Wang X., Ma G., Li Q. (2024). Activation of Cannabinoid Type 2 Receptor in Microglia Reduces Neuroinflammation through Inhibiting Aerobic Glycolysis to Relieve Hypertension. Biomolecules.

[60] Zou L., Xu X., Wang Y., Lin F., Zhang C., Liu R., Hou X., Wang J., Jiang X., Zhang Q. (2024). Neonatal Exposure to Polystyrene Nanoplastics Impairs Microglia-Mediated Synaptic Pruning and Causes Social Behavioral Defects in Adulthood. Environ. Sci. Technol..

[61] He D., Xu H., Zhang H., Tang R., Lan Y., Xing R., Li S., Christian E., Hou Y., Lorello P. (2022). Disruption of the IL-33-ST2-AKT signaling axis impairs neurodevelopment by inhibiting microglial metabolic adaptation and phagocytic function. Immunity.

[62] Tagliatti E., Desiato G., Mancinelli S., Bizzotto M., Gagliani M.C., Faggiani E., Hernandez-Soto R., Cugurra A., Poliseno P., Miotto M. (2024). Trem2 expression in microglia is required to maintain normal neuronal bioenergetics during development. Immunity.

[63] Wei W., Zhang L., Xin W., Pan Y., Tatenhorst L., Hao Z., Gerner S.T., Huber S., Juenemann M., Butz M. (2024). TREM2 regulates microglial lipid droplet formation and represses post-ischemic brain injury. Biomed. Pharmacother..

[64] Chausse B., Kakimoto P.A., Kann O. (2021). Microglia and lipids: How metabolism controls brain innate immunity. Semin. Cell Dev. Biol..

[65] Park D.S., Kozaki T., Tiwari S.K., Moreira M., Khalilnezhad A., Torta F., Olivie N., Thiam C.H., Liani O., Silvin A. (2023). iPS-cell-derived microglia promote brain organoid maturation via cholesterol transfer. Nature.

[66] Yang X., Chen Y.H., Liu L., Gu Z., You Y., Hao J.R., Sun N., Gao C. (2024). Regulation of glycolysis-derived L-lactate production in astrocytes rescues the memory deficits and Abeta burden in early Alzheimer’s disease models. Pharmacol. Res..

[67] Bakina O., Kettenmann H., Nolte C. (2022). Microglia form satellites with different neuronal subtypes in the adult murine central nervous system. J. Neurosci. Res..

[68] Makarava N., Safadi T., Bocharova O., Mychko O., Pandit N.P., Molesworth K., Baiardi S., Zhang L., Parchi P., Baskakov I.V. (2024). Reactive microglia partially envelop viable neurons in prion diseases. J. Clin. Investig..

[69] Haruwaka K., Ying Y., Liang Y., Umpierre A.D., Yi M.H., Kremen V., Chen T., Xie T., Qi F., Zhao S. (2024). Microglia enhance post-anesthesia neuronal activity by shielding inhibitory synapses. Nat. Neurosci..

[70] Tremblay M.E., Lowery R.L., Majewska A.K. (2010). Microglial interactions with synapses are modulated by visual experience. PLoS Biol..

[71] Di Palma M., Catalano M., Serpe C., De Luca M., Monaco L., Kunzelmann K., Limatola C., Conti F., Fattorini G. (2023). Lipopolysaccharide augments microglial GABA uptake by increasing GABA transporter-1 trafficking and bestrophin-1 expression. Glia.

[72] Broome S.T., Fisher T., Faiz A., Keay K.A., Musumeci G., Al-Badri G., Castorina A. (2021). Assessing the Anti-Inflammatory Activity of the Anxiolytic Drug Buspirone Using CRISPR-Cas9 Gene Editing in LPS-Stimulated BV-2 Microglial Cells. Cells.

[73] Bravo J., Ribeiro I., Terceiro A.F., Andrade E.B., Portugal C.C., Lopes I.M., Azevedo M.M., Sousa M., Lopes C.D.F., Lobo A.C. (2022). Neuron-Microglia Contact-Dependent Mechanisms Attenuate Methamphetamine-Induced Microglia Reactivity and Enhance Neuronal Plasticity. Cells.

[74] Cao X.W., Yang H., Liu X.M., Lou S.Y., Kong L.P., Rong L.Q., Shan J.J., Xu Y., Zhang Q.X. (2023). Blocking postsynaptic density-93 binding to C-X3-C motif chemokine ligand 1 promotes microglial phenotypic transformation during acute ischemic stroke. Neural Regen. Res..

[75] Scheiblich H., Eikens F., Wischhof L., Opitz S., Jüngling K., Cserép C., Schmidt S.V., Lambertz J., Bellande T., Pósfai B. (2024). Microglia rescue neurons from aggregate-induced neuronal dysfunction and death through tunneling nanotubes. Neuron.

[76] Picciotto M.R., Higley M.J., Mineur Y.S. (2012). Acetylcholine as a neuromodulator: Cholinergic signaling shapes nervous system function and behavior. Neuron.

[77] Waldhoer M., Bartlett S.E., Whistler J.L. (2004). Opioid receptors. Annu. Rev. Biochem..

[78] Ohtake J., Kaneumi S., Tanino M., Kishikawa T., Terada S., Sumida K., Masuko K., Ohno Y., Kita T., Iwabuchi S. (2015). Neuropeptide signaling through neurokinin-1 and neurokinin-2 receptors augments antigen presentation by human dendritic cells. J. Allergy Clin. Immunol..

[79] Moliner R., Girych M., Brunello C.A., Kovaleva V., Biojone C., Enkavi G., Antenucci L., Kot E.F., Goncharuk S.A., Kaurinkoski K. (2023). Psychedelics promote plasticity by directly binding to BDNF receptor TrkB. Nat. Neurosci..

[80] Pincelli C. (2017). p75 Neurotrophin Receptor in the Skin: Beyond Its Neurotrophic Function. Front. Med..

[81] Zipp F., Bittner S., Schafer D.P. (2023). Cytokines as emerging regulators of central nervous system synapses. Immunity.

[82] Fonseca M.I., Zhou J., Botto M., Tenner A.J. (2004). Absence of C1q leads to less neuropathology in transgenic mouse models of Alzheimer’s disease. J. Neurosci..

[83] Harland M., Torres S., Liu J., Wang X. (2020). Neuronal Mitochondria Modulation of LPS-Induced Neuroinflammation. J. Neurosci..

[84] Han B., Jiang W., Liu H., Wang J., Zheng K., Cui P., Feng Y., Dang C., Bu Y., Wang Q.M. (2020). Upregulation of neuronal PGC-1alpha ameliorates cognitive impairment induced by chronic cerebral hypoperfusion. Theranostics.

[85] Bae J.Y., Jacquemyn J., Ioannou M.S. (2024). Neuronal AMPK regulates lipid transport to microglia. Trends Cell Biol..

[86] Ronzano R., Roux T., Thetiot M., Aigrot M.S., Richard L., Lejeune F.X., Mazuir E., Vallat J.M., Lubetzki C., Desmazieres A. (2021). Microglia-neuron interaction at nodes of Ranvier depends on neuronal activity through potassium release and contributes to remyelination. Nat. Commun..

[87] Scheiblich H., Dansokho C., Mercan D., Schmidt S.V., Bousset L., Wischhof L., Eikens F., Odainic A., Spitzer J., Griep A. (2021). Microglia jointly degrade fibrillar alpha-synuclein cargo by distribution through tunneling nanotubes. Cell.

[88] Chakraborty R., Nonaka T., Hasegawa M., Zurzolo C. (2023). Tunnelling nanotubes between neuronal and microglial cells allow bi-directional transfer of alpha-Synuclein and mitochondria. Cell Death Dis..

[89] Xu J., Li Y., Novak C., Lee M., Yan Z., Bang S., McGinnis A., Chandra S., Zhang V., He W. (2026). Mitochondrial transfer from glia to neurons protects against peripheral neuropathy. Nature.

[90] Cserep C., Posfai B., Lenart N., Fekete R., Laszlo Z.I., Lele Z., Orsolits B., Molnar G., Heindl S., Schwarcz A.D. (2020). Microglia monitor and protect neuronal function through specialized somatic purinergic junctions. Science.

[91] Hughes A.N., Appel B. (2020). Microglia phagocytose myelin sheaths to modify developmental myelination. Nat. Neurosci..

[92] Azoulay E., Zuber J., Bousfiha A.A., Long Y., Tan Y., Luo S., Essafti M., Annane D. (2024). Complement system activation: Bridging physiology, pathophysiology, and therapy. Intensive Care Med..

[93] Scott-Hewitt N., Mahoney M., Huang Y., Korte N., Yvanka de Soysa T., Wilton D.K., Knorr E., Mastro K., Chang A., Zhang A. (2024). Microglial-derived C1q integrates into neuronal ribonucleoprotein complexes and impacts protein homeostasis in the aging brain. Cell.

[94] Harrison J.K., Jiang Y., Chen S., Xia Y., Maciejewski D., McNamara R.K., Streit W.J., Salafranca M.N., Adhikari S., Thompson D.A. (1998). Role for neuronally derived fractalkine in mediating interactions between neurons and CX3CR1-expressing microglia. Proc. Natl. Acad. Sci. USA.

[95] Kim K.W., Vallon-Eberhard A., Zigmond E., Farache J., Shezen E., Shakhar G., Ludwig A., Lira S.A., Jung S. (2011). In vivo structure/function and expression analysis of the CX3C chemokine fractalkine. Blood.

[96] Biber K., Neumann H., Inoue K., Boddeke H.W. (2007). Neuronal ‘On’ and ‘Off’ signals control microglia. Trends Neurosci..

[97] Cao P., Chen C., Liu A., Shan Q., Zhu X., Jia C., Peng X., Zhang M., Farzinpour Z., Zhou W. (2021). Early-life inflammation promotes depressive symptoms in adolescence via microglial engulfment of dendritic spines. Neuron.

[98] Chen X.T., Chen L.P., Fan L.J., Kan H.M., Wang Z.Z., Qian B., Pan Z.Q., Shen W. (2023). Microglial P2Y12 Signaling Contributes to Cisplatin-induced Pain Hypersensitivity via IL-18-mediated Central Sensitization in the Spinal Cord. J. Pain..

[99] He Y., Liu T., He Q., Ke W., Li X., Du J., Deng S., Shu Z., Wu J., Yang B. (2023). Microglia facilitate and stabilize the response to general anesthesia via modulating the neuronal network in a brain region-specific manner. Elife.

[100] Badimon A., Strasburger H.J., Ayata P., Chen X., Nair A., Ikegami A., Hwang P., Chan A.T., Graves S.M., Uweru J.O. (2020). Negative feedback control of neuronal activity by microglia. Nature.

[101] Umpierre A.D., Wu L.J. (2021). How microglia sense and regulate neuronal activity. Glia.

[102] Ferro A., Auguste Y.S.S., Cheadle L. (2021). Microglia, Cytokines, and Neural Activity: Unexpected Interactions in Brain Development and Function. Front. Immunol..

[103] Miyamoto A., Wake H., Ishikawa A.W., Eto K., Shibata K., Murakoshi H., Koizumi S., Moorhouse A.J., Yoshimura Y., Nabekura J. (2016). Microglia contact induces synapse formation in developing somatosensory cortex. Nat. Commun..

[104] Nebeling F.C., Poll S., Justus L.C., Steffen J., Keppler K., Mittag M., Fuhrmann M. (2023). Microglial motility is modulated by neuronal activity and correlates with dendritic spine plasticity in the hippocampus of awake mice. Elife.

[105] Nguyen P.T., Dorman L.C., Pan S., Vainchtein I.D., Han R.T., Nakao-Inoue H., Taloma S.E., Barron J.J., Molofsky A.B., Kheirbek M.A. (2020). Microglial Remodeling of the Extracellular Matrix Promotes Synapse Plasticity. Cell.

[106] Stogsdill J.A., Kim K., Binan L., Farhi S.L., Levin J.Z., Arlotta P. (2022). Pyramidal neuron subtype diversity governs microglia states in the neocortex. Nature.

[107] Pinto M.J., Bizien L., Fabre J.M.J., Ethukanovic N., Lepetz V., Henderson F., Pujol M., Sala R.W., Tarpin T., Popa D. (2024). Microglial TNFalpha controls daily changes in synaptic GABAARs and sleep slow waves. J. Cell Biol..

[108] Guo H., Chen L.Q., Zou Z.R., Cheng S., Hu Y., Mao L., Tian H., Mei X.F. (2024). Zinc remodels mitochondrial network through SIRT3/Mfn2-dependent mitochondrial transfer in ameliorating spinal cord injury. Eur. J. Pharmacol..

[109] Liu W., Qi Z., Li W., Liang J., Zhao L., Shi Y. (2022). M1 Microglia Induced Neuronal Injury on Ischemic Stroke via Mitochondrial Crosstalk between Microglia and Neurons. Oxid. Med. Cell. Longev..

[110] Pereira-Santos A.R., Candeias E., Magalhaes J.D., Empadinhas N., Esteves A.R., Cardoso S.M. (2024). Neuronal control of microglia through the mitochondria. Biochim. Biophys. Acta Mol. Basis Dis..

[111] Falzoni S., Vultaggio-Poma V., Chiozzi P., Tarantini M., Adinolfi E., Boldrini P., Giuliani A.L., Morciano G., Tang Y., Gorecki D.C. (2024). The P2X7 Receptor is a Master Regulator of Microparticle and Mitochondria Exchange in Mouse Microglia. Function.

[112] Gabrielli M., Raffaele S., Fumagalli M., Verderio C. (2022). The multiple faces of extracellular vesicles released by microglia: Where are we 10 years after?. Front. Cell. Neurosci..

[113] Arvanitaki E.S., Goulielmaki E., Gkirtzimanaki K., Niotis G., Tsakani E., Nenedaki E., Rouska I., Kefalogianni M., Xydias D., Kalafatakis I. (2024). Microglia-derived extracellular vesicles trigger age-related neurodegeneration upon DNA damage. Proc. Natl. Acad. Sci. USA.

[114] Peng H., Harvey B.T., Richards C.I., Nixon K. (2021). Neuron-Derived Extracellular Vesicles Modulate Microglia Activation and Function. Biology.

[115] Xin D., Li T., Zhao Y., Guo X., Gai C., Jiang Z., Yu S., Cheng J., Song Y., Cheng Y. (2024). MiR-100-5p-rich small extracellular vesicles from activated neuron to aggravate microglial activation and neuronal activity after stroke. J. Nanobiotechnology.

[116] Xian X., Cai L.L., Li Y., Wang R.C., Xu Y.H., Chen Y.J., Xie Y.H., Zhu X.L., Li Y.F. (2022). Neuron secrete exosomes containing miR-9-5p to promote polarization of M1 microglia in depression. J. Nanobiotechnology.

[117] Yin Z., Han Z., Hu T., Zhang S., Ge X., Huang S., Wang L., Yu J., Li W., Wang Y. (2020). Neuron-derived exosomes with high miR-21-5p expression promoted polarization of M1 microglia in culture. Brain Behav. Immun..

[118] Chausse B., Malorny N., Lewen A., Poschet G., Berndt N., Kann O. (2024). Metabolic flexibility ensures proper neuronal network function in moderate neuroinflammation. Sci. Rep..

[119] Bernier L.P., York E.M., Kamyabi A., Choi H.B., Weilinger N.L., MacVicar B.A. (2020). Microglial metabolic flexibility supports immune surveillance of the brain parenchyma. Nat. Commun..

[120] Frosch M., Shimizu T., Wogram E., Amann L., Gruber L., Groisman A.I., Fliegauf M., Schwabenland M., Chhatbar C., Zechel S. (2025). Microglia-neuron crosstalk through Hex-GM2-MGL2 maintains brain homeostasis. Nature.

[121] Hong S., Beja-Glasser V.F., Nfonoyim B.M., Frouin A., Li S., Ramakrishnan S., Merry K.M., Shi Q., Rosenthal A., Barres B.A. (2016). Complement and microglia mediate early synapse loss in Alzheimer mouse models. Science.

[122] Hu W., Chen M., Lin Y., Zhang H., Sun L., Shao W., Ye Y., Cheng Y., Zhou S., Hu P. (2025). Neuronal CD47 induces behavioral alterations and ameliorates microglial synaptic pruning in wild-type and Alzheimer’s mouse models. Cell Biosci..

[123] Gervais E., Iloun P., Martianova E., Goncalves Bessa A.C., Rivest S., Topolnik L. (2022). Structural analysis of the microglia-interneuron interactions in the CA1 hippocampal area of the APP/PS1 mouse model of Alzheimer’s disease. J. Comp. Neurol..

[124] Spangenberg E.E., Lee R.J., Najafi A.R., Rice R.A., Elmore M.R., Blurton-Jones M., West B.L., Green K.N. (2016). Eliminating microglia in Alzheimer’s mice prevents neuronal loss without modulating amyloid-beta pathology. Brain.

[125] Rao A., Chen N., Kim M.J., Blumenfeld J., Yip O., Liang Z., Shostak D., Hao Y., Nelson M.R., Koutsodendris N. (2025). Microglia depletion reduces human neuronal APOE4-related pathologies in a chimeric Alzheimer’s disease model. Cell Stem Cell.

[126] Haney M.S., Palovics R., Munson C.N., Long C., Johansson P.K., Yip O., Dong W., Rawat E., West E., Schlachetzki J.C.M. (2024). APOE4/4 is linked to damaging lipid droplets in Alzheimer’s disease microglia. Nature.

[127] Victor M.B., Leary N., Luna X., Meharena H.S., Scannail A.N., Bozzelli P.L., Samaan G., Murdock M.H., von Maydell D., Effenberger A.H. (2022). Lipid accumulation induced by APOE4 impairs microglial surveillance of neuronal-network activity. Cell Stem Cell.

[128] Ulland T.K., Song W.M., Huang S.C., Ulrich J.D., Sergushichev A., Beatty W.L., Loboda A.A., Zhou Y., Cairns N.J., Kambal A. (2017). TREM2 Maintains Microglial Metabolic Fitness in Alzheimer’s Disease. Cell.

[129] Austad S.N., Ballinger S., Buford T.W., Carter C.S., Smith D.L., Darley-Usmar V., Zhang J. (2022). Targeting whole body metabolism and mitochondrial bioenergetics in the drug development for Alzheimer’s disease. Acta Pharm. Sin. B.

[130] Zhong X., Gong S., Meng L., Yao W., Du K., Jiao L., Ma G., Liang J., Wei B., Jin X. (2024). Cordycepin Modulates Microglial M2 Polarization Coupled with Mitochondrial Metabolic Reprogramming by Targeting HKII and PDK2. Adv. Sci..

[131] Kim C., Cho E.D., Kim H.K., You S., Lee H.J., Hwang D., Lee S.J. (2014). beta1-integrin-dependent migration of microglia in response to neuron-released alpha-synuclein. Exp. Mol. Med..

[132] Yun S.P., Kam T.I., Panicker N., Kim S., Oh Y., Park J.S., Kwon S.H., Park Y.J., Karuppagounder S.S., Park H. (2018). Block of A1 astrocyte conversion by microglia is neuroprotective in models of Parkinson’s disease. Nat. Med..

[133] Yuan J., Liu H., Zhang H., Wang T., Zheng Q., Li Z. (2022). Controlled Activation of TRPV1 Channels on Microglia to Boost Their Autophagy for Clearance of Alpha-Synuclein and Enhance Therapy of Parkinson’s Disease. Adv. Mater..

[134] Lu J., Wang C., Cheng X., Wang R., Yan X., He P., Chen H., Yu Z. (2022). A breakdown in microglial metabolic reprogramming causes internalization dysfunction of alpha-synuclein in a mouse model of Parkinson’s disease. J. Neuroinflammation.

[135] Cai R., Zhang Y., Simmering J.E., Schultz J.L., Li Y., Fernandez-Carasa I., Consiglio A., Raya A., Polgreen P.M., Narayanan N.S. (2019). Enhancing glycolysis attenuates Parkinson’s disease progression in models and clinical databases. J. Clin. Investig..

[136] Liu J., Liang Y., Meng Q., Chen J., Ma J., Zhu H., Cai L., Song N., Ding J., Fan Y. (2024). Antagonism of beta-arrestins in IL-4-driven microglia reactivity via the Samd4/mTOR/OXPHOS axis in Parkinson’s disease. Sci. Adv..

[137] Schirinzi T., Madeo G., Martella G., Maltese M., Picconi B., Calabresi P., Pisani A. (2016). Early synaptic dysfunction in Parkinson’s disease: Insights from animal models. Mov. Disord..

[138] Chen Z., Li Q., Wang K., Yang L., Jia Y., Gong Z. (2024). Brain Pericytes—Crucial Regulators of Neuroinflammation in Ischemic Stroke. Neuropharmacol. Ther..

[139] Cignarella F., Filipello F., Bollman B., Cantoni C., Locca A., Mikesell R., Manis M., Ibrahim A., Deng L., Benitez B.A. (2020). TREM2 activation on microglia promotes myelin debris clearance and remyelination in a model of multiple sclerosis. Acta Neuropathol..

[140] Dong Y., D’Mello C., Pinsky W., Lozinski B.M., Kaushik D.K., Ghorbani S., Moezzi D., Brown D., Melo F.C., Zandee S. (2021). Oxidized phosphatidylcholines found in multiple sclerosis lesions mediate neurodegeneration and are neutralized by microglia. Nat. Neurosci..

[141] Goulart M.T., Queiroz D.T.U., Ribeiro F.M. (2025). The Dual Role of Microglia in Multiple Sclerosis and its Implications for Diagnostics and Repair. Curr. Neuropharmacol..

[142] Bhargava P., Smith M.D., Mische L., Harrington E., Fitzgerald K.C., Martin K., Kim S., Reyes A.A., Gonzalez-Cardona J., Volsko C. (2020). Bile acid metabolism is altered in multiple sclerosis and supplementation ameliorates neuroinflammation. J. Clin. Investig..

[143] Sadeghdoust M., Das A., Kaushik D.K. (2024). Fueling neurodegeneration: Metabolic insights into microglia functions. J. Neuroinflammation.

[144] Schirmer L., Velmeshev D., Holmqvist S., Kaufmann M., Werneburg S., Jung D., Vistnes S., Stockley J.H., Young A., Steindel M. (2019). Neuronal vulnerability and multilineage diversity in multiple sclerosis. Nature.

[145] Creus-Muncunill J., Haure-Mirande J.V., Mattei D., Bons J., Ramirez A.V., Hamilton B.W., Corwin C., Chowdhury S., Schilling B., Ellerby L.M. (2024). TYROBP/DAP12 knockout in Huntington’s disease Q175 mice cell-autonomously decreases microglial expression of disease-associated genes and non-cell-autonomously mitigates astrogliosis and motor deterioration. J. Neuroinflammation.

[146] Gao C., Jiang J., Tan Y., Chen S. (2023). Microglia in neurodegenerative diseases: Mechanism and potential therapeutic targets. Signal Transduct. Target. Ther..

[147] Saba J., Couselo F.L., Bruno J., Carniglia L., Durand D., Lasaga M., Caruso C. (2022). Neuroinflammation in Huntington’s Disease: A Starring Role for Astrocyte and Microglia. Curr. Neuropharmacol..

[148] Palpagama T.H., Waldvogel H.J., Faull R.L.M., Kwakowsky A. (2019). The Role of Microglia and Astrocytes in Huntington’s Disease. Front. Mol. Neurosci..

[149] Wilton D.K., Mastro K., Heller M.D., Gergits F.W., Willing C.R., Fahey J.B., Frouin A., Daggett A., Gu X., Kim Y.A. (2023). Microglia and complement mediate early corticostriatal synapse loss and cognitive dysfunction in Huntington’s disease. Nat. Med..

[150] Lu Y.S., Hung W.C., Hsieh Y.T., Tsai P.Y., Tsai T.H., Fan H.H., Chang Y.G., Cheng H.K., Huang S.Y., Lin H.C. (2024). Equilibrative nucleoside transporter 3 supports microglial functions and protects against the progression of Huntington’s disease in the mouse model. Brain Behav. Immun..

[151] Crapser J.D., Ochaba J., Soni N., Reidling J.C., Thompson L.M., Green K.N. (2020). Microglial depletion prevents extracellular matrix changes and striatal volume reduction in a model of Huntington’s disease. Brain.

[152] Prieto G.A., Cotman C.W. (2022). Early bioenergetic and autophagy impairments at the Parkinson’s disease synapse. Brain.

[153] Cheng J., Zhang R., Xu Z., Ke Y., Sun R., Yang H., Zhang X., Zhen X., Zheng L.T. (2021). Early glycolytic reprogramming controls microglial inflammatory activation. J. Neuroinflammation.

[154] Guo M., Wang J., Zhao Y., Feng Y., Han S., Dong Q., Cui M., Tieu K. (2020). Microglial exosomes facilitate alpha-synuclein transmission in Parkinson’s disease. Brain.

[155] Merino-Galan L., Jimenez-Urbieta H., Zamarbide M., Rodriguez-Chinchilla T., Belloso-Iguerategui A., Santamaria E., Fernandez-Irigoyen J., Aiastui A., Doudnikoff E., Bezard E. (2022). Striatal synaptic bioenergetic and autophagic decline in premotor experimental parkinsonism. Brain.

[156] Distefano-Gagne F., Bitarafan S., Lacroix S., Gosselin D. (2023). Roles and regulation of microglia activity in multiple sclerosis: Insights from animal models. Nat. Rev. Neurosci..

[157] Vermersch P., Airas L., Berger T., Deisenhammer F., Grigoriadis N., Hartung H.P., Magyari M., Popescu V., Pozzilli C., Pugliatti M. (2025). The role of microglia in multiple sclerosis: Implications for treatment with Bruton’s tyrosine kinase inhibitors. Front. Immunol..

[158] Barros C., Alberro A., Fernandes A. (2024). Microglia and Immune cells interactions in multiple sclerosis cognitive impairment: A postmortem study. J. Neuroinflammation.

[159] Zelic M., Pontarelli F., Woodworth L., Zhu C., Mahan A., Ren Y., LaMorte M., Gruber R., Keane A., Loring P. (2021). RIPK1 activation mediates neuroinflammation and disease progression in multiple sclerosis. Cell Rep..

[160] Ganz T., Fainstein N., Theotokis P., Elgavish S., Vardi-Yaakov O., Lachish M., Sofer L., Zveik O., Grigoriadis N., Ben-Hur T. (2024). Targeting CNS myeloid infiltrates provides neuroprotection in a progressive multiple sclerosis model. Brain Behav. Immun..

[161] Gao C., Lin Z., Chen Y., Gan R., Wang K., He X.X., Han H. (2025). The Spectrum of Microglia: Decoding Heterogeneity and Plasticity for Therapeutic Gain in Neurological Disorders. Neuropharmacol. Ther..

[162] Kimura K., Subramanian A., Yin Z., Khalilnezhad A., Wu Y., He D., Dixon K.O., Chitta U.K., Ding X., Adhikari N. (2025). Immune checkpoint TIM-3 regulates microglia and Alzheimer’s disease. Nature.

[163] Chen X., Huang Y., Huang L., Huang Z., Hao Z.Z., Xu L., Xu N., Li Z., Mou Y., Ye M. (2024). A brain cell atlas integrating single-cell transcriptomes across human brain regions. Nat. Med..

[164] Scheepstra K.W.F., Mizee M.R., van Scheppingen J., Adelia A., Wever D.D., Mason M.R.J., Dubbelaar M.L., Hsiao C.C., Eggen B.J.L., Hamann J. (2023). Microglia Transcriptional Profiling in Major Depressive Disorder Shows Inhibition of Cortical Gray Matter Microglia. Biol. Psychiatry.

[165] Böttcher C., Fernández-Zapata C., Snijders G.J.L., Schlickeiser S., Sneeboer M.A.M., Kunkel D., De Witte L.D., Priller J. (2020). Single-cell mass cytometry of microglia in major depressive disorder reveals a non-inflammatory phenotype with increased homeostatic marker expression. Transl. Psychiatry.

[166] Xia X., Li K., Zou W., Wang L. (2025). The central role of microglia in major depressive disorder and its potential as a therapeutic target. Front. Behav. Neurosci..

[167] Li B., Yang W., Ge T., Wang Y., Cui R. (2022). Stress induced microglial activation contributes to depression. Pharmacol. Res..

[168] Li J., Seidlitz J., Suckling J., Fan F., Ji G.J., Meng Y., Yang S., Wang K., Qiu J., Chen H. (2021). Cortical structural differences in major depressive disorder correlate with cell type-specific transcriptional signatures. Nat. Commun..

[169] Wang H., He Y., Sun Z., Ren S., Liu M., Wang G., Yang J. (2022). Microglia in depression: An overview of microglia in the pathogenesis and treatment of depression. J. Neuroinflammation.

[170] Hellwig S., Brioschi S., Dieni S., Frings L., Masuch A., Blank T., Biber K. (2016). Altered microglia morphology and higher resilience to stress-induced depression-like behavior in CX3CR1-deficient mice. Brain Behav. Immun..

[171] Moloney E.B., Moskites A., Ferrari E.J., Isacson O., Hallett P.J. (2018). The glycoprotein GPNMB is selectively elevated in the substantia nigra of Parkinson’s disease patients and increases after lysosomal stress. Neurobiol. Dis..

[172] Badanjak K., Fixemer S., Smajić S., Skupin A., Grünewald A. (2021). The Contribution of Microglia to Neuroinflammation in Parkinson’s Disease. Int. J. Mol. Sci..

[173] Lv Q.K., Tao K.X., Wang X.B., Yao X.Y., Pang M.Z., Liu J.Y., Wang F., Liu C.F. (2023). Role of α-synuclein in microglia: Autophagy and phagocytosis balance neuroinflammation in Parkinson’s disease. Inflamm. Res..

[174] Sirerol-Piquer M.S., Perez-Villalba A., Duart-Abadia P., Belenguer G., Gómez-Pinedo U., Blasco-Chamarro L., Carrillo-Barberà P., Pérez-Cañamás A., Navarro-Garrido V., Dehay B. (2025). Age-dependent progression from clearance to vulnerability in the early response of periventricular microglia to α-synuclein toxic species. Mol. Neurodegener..

[175] Geirsdottir L., David E., Keren-Shaul H., Weiner A., Bohlen S.C., Neuber J., Balic A., Giladi A., Sheban F., Dutertre C.A. (2019). Cross-Species Single-Cell Analysis Reveals Divergence of the Primate Microglia Program. Cell.

[176] Galatro T.F., Holtman I.R., Lerario A.M., Vainchtein I.D., Brouwer N., Sola P.R., Veras M.M., Pereira T.F., Leite R.E.P., Möller T. (2017). Transcriptomic analysis of purified human cortical microglia reveals age-associated changes. Nat. Neurosci..

[177] Hasselmann J., Blurton-Jones M. (2020). Human iPSC-derived microglia: A growing toolset to study the brain’s innate immune cells. Glia.

[178] Mancuso R., Fattorelli N., Martinez-Muriana A., Davis E., Wolfs L., Van Den Daele J., Geric I., Premereur J., Polanco P., Bijnens B. (2024). Xenografted human microglia display diverse transcriptomic states in response to Alzheimer’s disease-related amyloid-β pathology. Nat. Neurosci..

[179] Dias D., Portugal C.C., Relvas J., Socodato R. (2025). From Genetics to Neuroinflammation: The Impact of ApoE4 on Microglial Function in Alzheimer’s Disease. Cells.

[180] Xu R., Boreland A.J., Li X., Erickson C., Jin M., Atkins C., Pang Z.P., Daniels B.P., Jiang P. (2021). Developing human pluripotent stem cell-based cerebral organoids with a controllable microglia ratio for modeling brain development and pathology. Stem Cell Rep..

[181] Zhang W., Jiang J., Xu Z., Yan H., Tang B., Liu C., Chen C., Meng Q. (2023). Microglia-containing human brain organoids for the study of brain development and pathology. Mol. Psychiatry.

